# Of Mice, Birds, and Men: The Mouse Ultrasonic Song System Has Some Features Similar to Humans and Song-Learning Birds

**DOI:** 10.1371/journal.pone.0046610

**Published:** 2012-10-10

**Authors:** Gustavo Arriaga, Eric P. Zhou, Erich D. Jarvis

**Affiliations:** 1 Department of Neurobiology, Howard Hughes Medical Institute, Duke University Medical Center, Durham, North Carolina, United States of America; 2 Tulane University School of Medicine, New Orleans, Louisiana, United States of America; Northwestern University, United States of America

## Abstract

Humans and song-learning birds communicate acoustically using learned vocalizations. The characteristic features of this social communication behavior include vocal control by forebrain motor areas, a direct cortical projection to brainstem vocal motor neurons, and dependence on auditory feedback to develop and maintain learned vocalizations. These features have so far not been found in closely related primate and avian species that do not learn vocalizations. Male mice produce courtship ultrasonic vocalizations with acoustic features similar to songs of song-learning birds. However, it is assumed that mice lack a forebrain system for vocal modification and that their ultrasonic vocalizations are innate. Here we investigated the mouse song system and discovered that it includes a motor cortex region active during singing, that projects directly to brainstem vocal motor neurons and is necessary for keeping song more stereotyped and on pitch. We also discovered that male mice depend on auditory feedback to maintain some ultrasonic song features, and that sub-strains with differences in their songs can match each other's pitch when cross-housed under competitive social conditions. We conclude that male mice have some limited vocal modification abilities with at least some neuroanatomical features thought to be unique to humans and song-learning birds. To explain our findings, we propose a continuum hypothesis of vocal learning.

## Introduction

**Figure 1:**
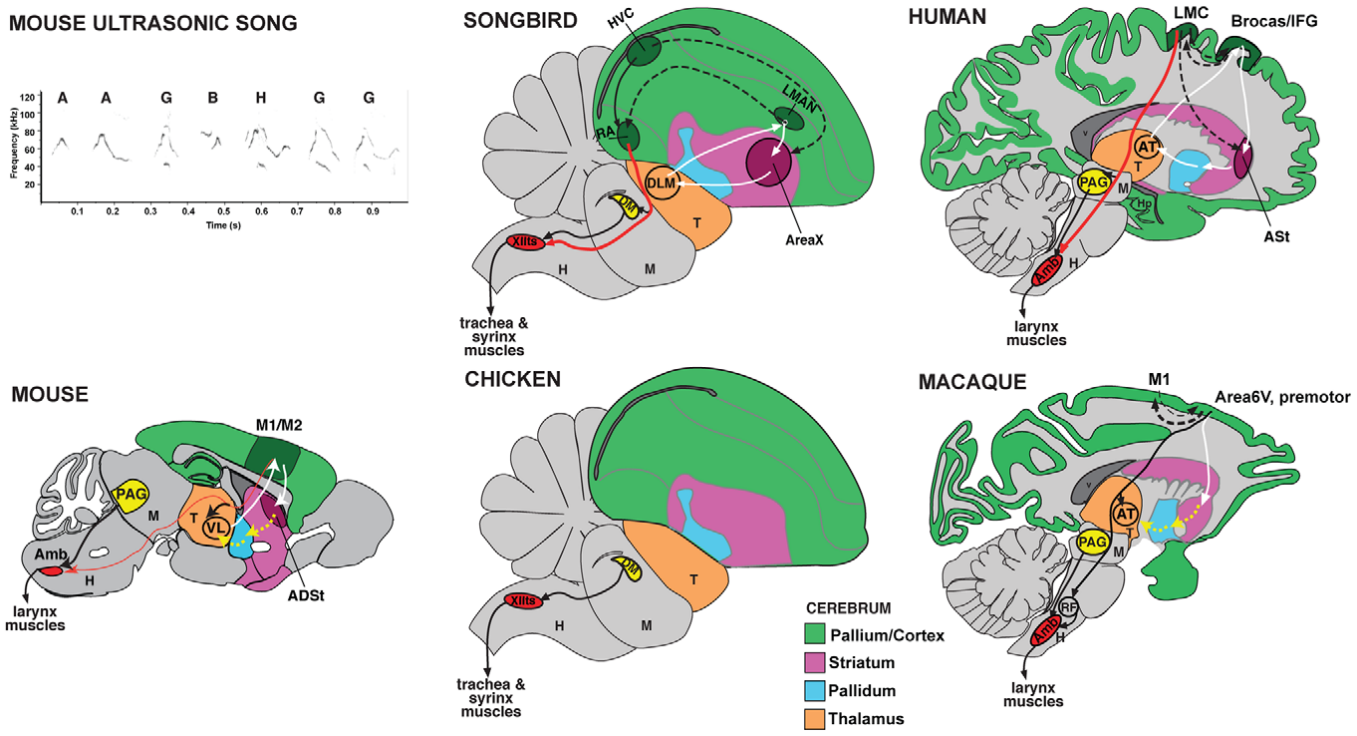
Brain systems for vocalization in birds and mammals. **A,** Typical ultrasonic song segment (sonogram) of a male B6D2F1/J (BxD) mouse produced in response to presentation of female urine. Multiple distinct syllables (letters) are produced in long sequences (sometimes over 30 sec), but only 1 second is shown so that the frequency contours and nonlinearities of individual units can be resolved. The sonogram was generated from Audio S1. **B–C**, Summary diagrams of vocal learning systems in songbirds and proposed pathway in humans [6]. Red arrows, the direct forebrain projection to vocal motor neurons in the brainstem (RA to XIIts in song learning birds; Laryngeal motor cortex [LMC] to Amb in humans) [6], [9], [13], [18]. White lines, anterior forebrain premotor circuits, including cortico-striatal-thalamic loops. Dashed lines, connections between the anterior forebrain and posterior vocal motor circuits. **D–E**, The direct cortico-bulbar projection is said to be absent in vocal non-learners such as chickens and monkeys. Monkeys possess an indirect cortical pathway to Amb [22], but this circuit does not appear to influence programming of vocalizations [9]. **F**, Summary diagram of mouse song system connectivity discovered in this study. Two pathways converge on Amb: one originating from the periaqueductal grey (PAG) and one from M1 (red arrow) similar to humans (C). Yellow lines indicate proposed connections for cortico-striatal-thalamic loop that need to be tested. Auditory input is not shown. All diagrams show the sagittal view. Abbreviations: ADSt, anterior dorsal striatum; Amb, nucleus ambiguous; Area 6V, ventral part of Area 6 premotor cortex; Area X, a song nucleus of the striatum; ASt, anterior striatum; AT, anterior thalamus; DLM, dorsalateral nucleus of the mesencephalon; DM, dorsal medial nucleus of the midbrain; H, hindbrain; Hp, hippocampus; HVC – letter based name; IFG, inferior frontal gyrus; LMAN, lateral magnocellular nucleus of the anterior nidopallium; LMC, laryngeal motor cortex; M, midbrain; M1, primary motor cortex; M2, secondary motor cortex; nXIIts, 12th tracheosynringeal motor neurons; PAG, periaqueductal grey; RA, robust nucleus of the arcopallium; RF, reticular formation; T, thalamus; VL, ventral lateral nucleus of the thalamus.

Male mice are known to produce ultrasonic vocalizations (USVs) in a mating context, and these have been assumed to be exclusively innate [1]. A recent seminal study by Holy and Guo [2] demonstrated that features of male mouse USVs have some characteristics of song behaviors observed in songbirds (Figure 1A; sound recording in Audio S1). These features include the following: melodic structure of the vocalizations; sequential vocal structure unlike ‘calls’ which by definition are isolated or repeated syllables of typically one type; syllables produced in a non-random sequence with repeated motifs; and individual differences in repertoire composition. For these and other reasons, Holy and Guo called these male USVs ‘mouse songs’ [2]. We note that this designation does not imply learning, as songs or calls of different species can be learned or innate [3], [4]. However, the discovery of USV song in mice opened the question of whether mice share any behavioral and neural mechanisms for song production and learning with the set of rare vocal learning species, which includes three groups of birds (songbirds, parrots, hummingbirds) and several groups of mammals (humans, cetaceans [dolphins and whales], bats, elephants, and pinnipeds [sea lions and seals]) [5]–[7].

Vocal learning is the ability to modify the spectral and syntactic composition of vocalizations generated by the vocal organ (larynx in mammals or syrinx in birds). This ability is a critical substrate for human speech and is well studied in songbirds and parrots, two groups of birds with a remarkable capacity for vocal mimicry using a process similar to human speech acquisition [6], [8]. Underlying this process in humans and song-learning birds are specialized forebrain circuits so far not found in species that produce only innate vocalizations, despite decades of searching for them (Figure 1B–E) [6], [9]. Even closely related non-human primate species reportedly lack the behavioral and neural elements classically associated with vocal learning [5], [9], although they can make small changes to innately specified vocalizations [10]. Mice have been assumed to be members of the vocal non-learning category [1], [6], [11], but this had not been tested when we began our study. Here we asked whether major features considered unique to vocal learners are present in mice. We identified part of the neural system for courtship USVs in male mice and show that both the brain and behavior display some features characteristic of humans and song-learning birds. Based on these results we suggest that vocal learning among extant species may not be a dichotomous trait as commonly believed, but distributed along a spectrum of categories, which we call a continuum hypothesis of vocal learning [4].

## Results

### The mouse song system: activation of motor cortex and striatum

A common feature found in vocal learning species tested to date (songbirds, parrots, hummingbirds, and humans) is dedicated cortico-striatal-thalamic circuits active during production of learned vocalizations [1], [6], [9], [12]. In contrast, vocalization-specific activity in vocal non-learning species has been convincingly demonstrated in limbic forebrain, midbrain and brainstem circuits [9], [13]. Such vocalization-activated brain regions have typically been identified with functional neuroimaging techniques during speech production in humans [14], and with imaging, electrophysiological recordings, micro-stimulation, and behavioral molecular mapping of activity-dependent genes in non-human animals [6], [9]. To identify brain regions of the mouse USV system, we used a behavioral molecular mapping experimental design similar to that used to identify seven similar forebrain song nuclei among separate lineages of song-learning birds [12], [15]. Sexually experienced adult males of the same B6D2F1/J strain (abbreviated BxD) used by Holy and Guo [2] were isolated overnight and divided into four groups: Non-Singing (males stimulated with a non-sexual ethanol odorant); Hearing Only (males presented with playback of ultrasonic vocalizations); Hearing & Singing (males stimulated with female urine to sing ultrasonic songs); and Deaf-Singing (deafened males stimulated with female urine to sing ultrasonic songs). After 30 min, the animals were sacrificed and entire brains were assayed for behaviorally driven expression of the activity-dependent immediate early genes (IEG) egr1 and arc.

**Figure 2:**
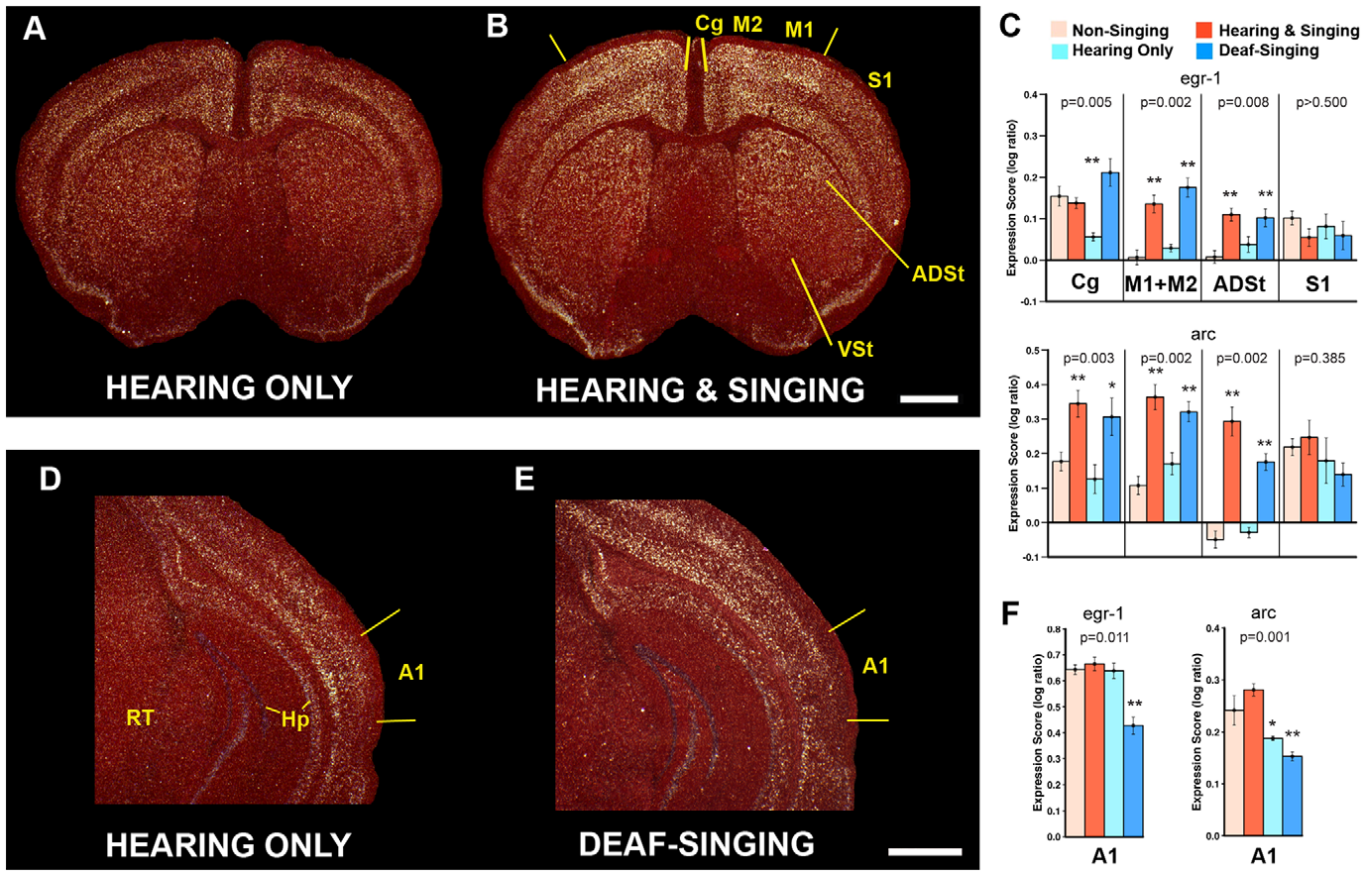
Behavioral-molecular mapping of mouse song system forebrain areas. **A–B**, Dark-field images of cresyl violet stained (red) coronal brain sections showing in-situ hybridization of singing-induced egr1 expression (white) in the forebrain of male mice. The Hearing Only animal heard playbacks of USVs for 30 min. The Hearing & Singing animal sang 4,304 syllables in 30 min. Yellow lines mark the edges of the motor region with singing-driven gene expression. **C**, egr-1 and arc expression scores (log10 ratios normalized to the ventral striatum, see methods) for the four groups in five brain regions. **D–E**, Primary auditory cortex (A1; one hemisphere) of animals from the Hearing Only and Deaf-Singing groups. **F**, egr-1 and arc expression scores in A1 normalized to the midbrain reticular nucleus (RT). Kruskal-Wallis H-tests were used to test for mean differences across all groups in each region (n = 5 per group; p values reported on graphs), followed by the Mann-Whitney U to directly test differences between each group relative to Non-Singing controls (* = p<0.05, ** = p<0.01). Data are reported as means ± s.e.m. Scale bars, 1 mm. Additional data and arc *in-situ* hybridizations are shown in Figure S1. Abbreviations: A1, primary auditory cortex; ADSt, anterior dorsal striatum; Cg, cingulate cortex; Hp, hippocampus; M1, primary motor cortex; M2, secondary motor cortex; RT, reticular nucleus; S1, primary somatosensory cortex; VSt, ventral striatum.

We found that relative to Hearing Only USV controls, animals of the Hearing & Singing group showed significantly higher egr1 mRNA expression in a visibly restricted ∼8500 µm^2^ cortical region around the level of the anterior commissure containing adjacent portions of the primary (M1) and secondary (M2) motor cortices, as well as an increase in the subjacent anterodorsal striatum (ADSt; Figure 2A–C). Outside of these boundaries, most of the cortex (including other areas of M1/M2) and striatum did not appear to differ between the two groups, independent of absolute expression levels. For example, the adjacent somatosensory cortex (S1) had variable egr1 expression among animals, and the ventral striatum (VSt) and the midbrain reticular nucleus (Rt) had visibly low expression in most, but expression levels were not significantly different between the Hearing Only and Hearing & Singing groups (Figure 2A–C; Figure S1E, S1F). The Hearing Only group did not show significantly higher egr1 expression in the M1, M2, and ADSt regions relative to Non-Singing controls (Figure 2C), suggesting that the induction in the Hearing & Singing group was probably not due to mice hearing themselves sing. Consistent with this interpretation, the Deaf-Singing animals still showed induced egr1 expression in M1, M2, and subjacent ADSt at levels similar to the Hearing & Singing group (Figure 2C), but significantly reduced egr1 expression in the primary auditory cortex (A1) relative to all other groups (Figure 2D–F).

We noted basal egr1 expression in M1, M2, and subjacent ADSt of the Non-Singing and Hearing Only control groups (Figure 2A). To test if the expression was related to non-vocal motor behavior, we looked for a correlation between IEG expression and the amount of ambulatory movement recorded, but did not find a relationship (Figure S2A); even animals that sat still during most of the test session showed some basal expression (Figure S2A). Instead, the values separated best according to the animals that sang (higher expression) relative to those that did not (Figure S2A). The number of syllables produced was not correlated with egr1 expression (p>0.5; r^2^ = 0.0340; n = 10; simple linear regression), but differences were not expected because all mice from the singing groups sang a lot with little variation. The egr1 mRNA expression pattern was similar to arc, except that in the adjacent cingulate cortex (Cg), egr1 showed increased expression in all groups relative to the Hearing Only group and arc showed increased expression only in the singing groups (Figure 2A–C; Figure S1A–B). We do not have an explanation for this difference, except that different IEGs can have different sensitivity responses to neural activity.

These results suggest that male mice, like song-learning birds [12], [15], have motor cortical and striatal regions with motor-driven IEG expression during the production of songs in the absence of auditory feedback. The activated regions of mouse brain, however, are not discrete nuclei as in song-learning birds, and there is some basal expression without singing. These differences between species could be due to known differences in mammalian and avian brains [16]. Mammalian cortical cells are distributed in layers, and cells controlling different behaviors can be intermingled [17]; bird cortical-like pallial cells are organized as spatially segregated clusters [16].

### A direct cortical projection to vocal motor neurons

A common finding is that brain systems with direct cortico-bulbar connections to motor nuclei are associated with motor learning and fine motor control [17]. Consistent with this doctrine, humans and song-learning birds posses a direct connection from motor cortical areas (laryngeal motor cortex and arcopallial song nucleus, respectively) to the brainstem motor nuclei that control the vocal organ (nucleus ambiguus [Amb] and 12^th^ tracheosyringeal neurons [XIIts], respectively; Figure 1B, 1C) [6], [9], [13], [18], [19]. By contrast, these projections have yet to be found in innate-vocalizing species despite over 50 years of effort searching for them, particularly in vocal non-learning birds and non-human primates (Figure 1D, 1E) [9], [13], [20]–[22]. However, vocal non-learners can have direct connections for non-vocal motor learning pathways [17], from which vocal learning pathways have been proposed to have emerged [23]. Because of these findings, many researchers have proposed that the evolution of direct connections between cortical vocal premotor and brainstem vocal motor neurons may have been one of the key events that lead to the evolution of speech and song learning by allowing greater voluntary control over the fine structure of vocalizations [1], [6], [7], [9], [13], [14], [18], [19], [24]–[26] (Text S1). Although this key projection has not yet been searched for in non-human mammalian vocal learners (bats, dolphins, and elephants), mice have been assumed to lack it [1], [6]. We tested whether mice lack or possess a similar projection by beginning at the larynx and tracing back through premotor circuits with a retrograde trans-synaptic viral tracer.

**Figure 3:**
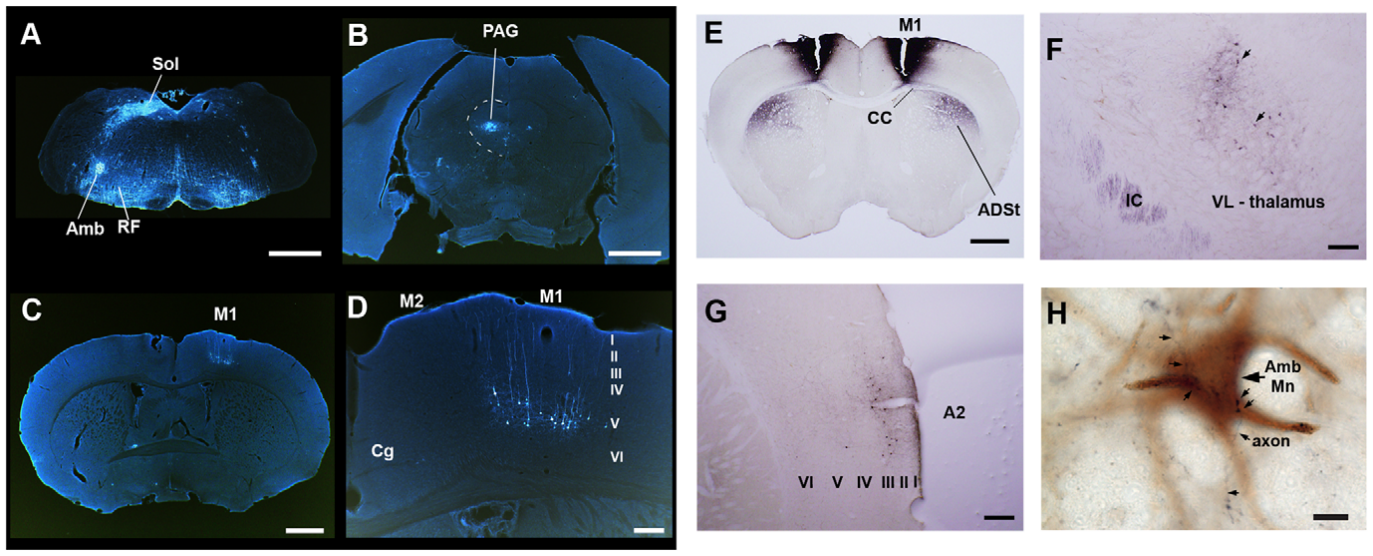
Mouse song system connectivity. **A**, Transynaptic PRV-Bartha expressing eGFP (white) in Amb from an injection in laryngeal muscles; tracer jumped to the surrounding reticular formation (RF) and solitary nucleus (Sol); color inverted from original brightfield image. **B**, Labeled cells bodies in the vocal part of the peri-aqueductal grey (PAG) of the same animal. **C**, Localized labeled layer V pyramidal neurons in the singing activated region of M1 of the same animal. **D**, Higher magnification of the cells in (C). **E**, Bilateral BDA injections (black) fill laryngeally connected M1 and reveal a dense projection to ADSt. **F**, M1 axons in the internal capsule (IC) with some terminations in VL of the thalamus; VL also has retrogradely filled neurons (arrows) that project to M1. **G**, Backfilled layer III cells of secondary auditory cortex (A2) from the same animal in (E). The auditory cortex region was verified with cytochrome oxidase label (not shown). **H**, Fine caliber M1 axons (black arrows) contact CTb-labeled laryngeal Amb motor neurons (MN; brown) from the same animal in (E). All sections are coronal. Scale bars: 1 mm for **A,C,E**; 200 µm for **B,D,G,H**; 10 µm for **H**. Abbreviations, the same as Figure 2 legend; additional abbreviations: CC, corpus collusm; Sol, solitary nucleus; IC, internal capsule; RF, reticular formation.

A recombinant pseudorabies virus (PRV-Bartha) expressing enhanced green fluorescent protein (eGFP) was injected into 2 of the 7 laryngeal muscles (cricothyroid and cricoarytenoid lateralis) of 12 male mice; PRV-Bartha only crosses functional synapses retrogradely through the sequence of synaptic connections away from the infection site [27], [28]. These two muscles are the most easily accessible using a ventral approach, and the cricothyroid muscle is likely involved in rapid pitch changes [29]. Consistent with known connectivity [30], neurons expressing eGFP were found in the ipsilateral vocal motor Amb neurons (Figure 3A). At approximately 90 hrs after injection, PRV-Bartha had spread from Amb to the surrounding reticular formation (RF), and other brainstem and midbrain nuclei with known direct connections to Amb and roles in the control of respiration and production of innate species-specific calls in mammals [9], including, respectively, the solitary nucleus (Sol) and central part of the periaqueductal grey (PAG) (Figure 3A–B). Interestingly, at this short latency we also observed a distinctly labeled contralateral population of layer V pyramidal neurons that co-localized with the same M1 motor cortex region that exhibited singing-driven IEG expression (Figure 3C–D vs Figure 2B). There were ∼306 labeled cells per hemisphere of each animal within an ∼840 µm anterior-posterior region of M1 (estimated from 102±16 cells in seven 40 µm thick sections per hemisphere, counting every third section). This number may represent a lower bound because we only injected tracer in 2 of the 7 laryngeal muscles; but it is in the range expected for layer V cortical muscle representation in non-human mammals [17]. Except for some isolated cells in layer III of the ipsilateral insular cortex (not shown), there were no other labeled cell clusters throughout the cortex. This pattern of cortical labeling was only observed together with second degree label in the midbrain PAG, in all 9 animals at the ∼90 hr survival time.

The above findings suggest that the M1 projection to Amb is direct, but does not prove it. To test whether it is direct, we made injections of biotinylated dextran amines (BDA) sufficiently large to encompass the singing-activated and PRV-backfilled region of M1 (n = 5 bilateral; n = 1 unilateral), and injected cholera toxin subunit b (CTb) into the laryngeal muscles to retrogradely label laryngeal motor neurons in a subset of animals (n = 3). We found that this portion of M1 projected robustly via the corpus callosum (CC) to a part of the ADSt in the striatum that displayed singing-driven IEG expression (Figure 3E vs Figure 2B). This region of M1 also connected via the internal capsule (IC) reciprocally to the ipsilateral ventral lateral nucleus of the thalamus (VL) (Figure 2F), and it was innervated by a distinct set of layer III neurons from the secondary auditory (A2) cortex (Figure 2G). Importantly, the same M1 cortical region sent descending axons to the brainstem, where a subset exited the medullary pyramids, extended laterally in the reticular formation and then terminated onto the primary dendrites and cell bodies of the backfilled CTb-positive motor neurons in Amb (Figure 3H; More examples in Figure S3A–C). The projection was sparse, with only about 1–2 axons per motor neuron and 1–3 axon bouton contact sites per soma. This sparseness and type of axon contact was similar to the projections seen from corticospinal projections to proprioceptive spinal cord neurons in rats when using BDA and CTb double labels [31]. We also found axons medially adjacent to nucleus ambiguous (Figure 3D), but there were many regions of the reticular formation without labeled axons (Figure S3E and not shown), indicating some specificity of the connection. This pattern of BDA-labeled axons in the forebrain and in Amb was seen in all 6 animals injected. A more detailed description of the connectivity and gene expression results is being prepared for a separate report. The combined findings suggest the presence in mice of a laryngeally connected M1 motor cortex that is functionally active in USV song behavior, that projects directly to brainstem vocal motor neurons and parts of the anterior striatum and thalamus, and receives input from the thalamus and secondary auditory cortex (Figure 1F). This pattern of connectivity is similar to known circuits in humans and song-learning birds (Figure 1B–C), but is much more sparse for the cortical to vocal motor neuron projection.

### Motor cortical pathway is required for modulating song

**Figure 4:**
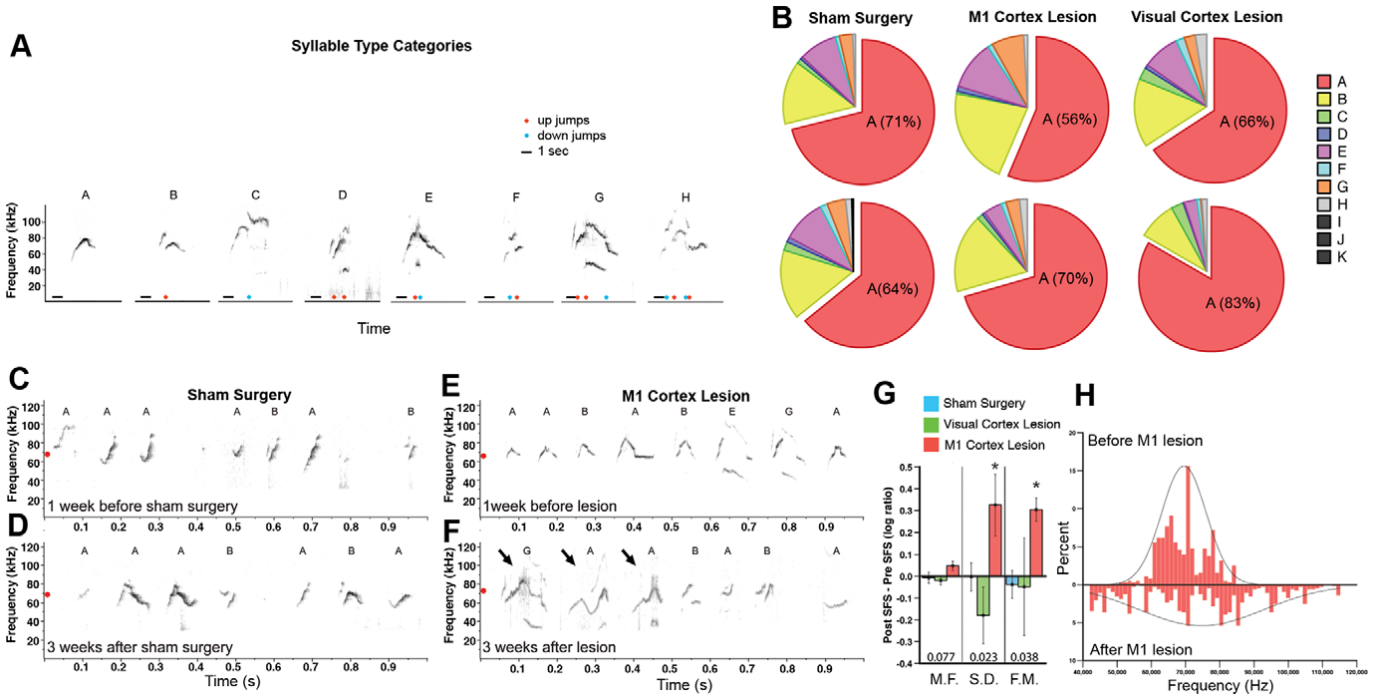
Song production following lesion of laryngeally connected motor cortex. **A,** Syllable category types from courtship USV of adult male BxD mice. A syllable is a series of one or more notes (continuous uninterrupted sound) and the corresponding sequence of instantaneous jumps (>10 kHz) in the dominant pitch [2]; blue dots - ‘Up’ jumps; red dots - ‘Down’ jumps. Because a jump is defined based on the instantaneous peak frequency, the harmonics in some notes are not considered for classification. Scale bar: 20 ms. **B**, Pie charts of syllable repertoire composition (categories in panel A) of male mice in each of the three surgery groups (n = 6 Sham surgery; n = 5 M1 Cortex Lesion; n = 4 Visual Cortex Lesion). **C–F**, Sonograms of male USVs before and after sham surgery or laryngeally connected M1 lesion (pitch-shifted recordings in **Audios S2–5**). Red dots, average pitch. Arrows point to examples of syllables with increased modulation relative to before M1 lesions. **G**, Spectral feature scores (SFS; expressed as log-ratio) for the mean frequency (M.F.) of the pitch, standard deviation (S.D.) of the pitch distribution, and frequency modulation (F.M.) for Type A syllables before and after surgery (* = p<0.05; Mann-Whitney U Test). Data are plotted as means ± s.e.m, from an average of 1731±381 s.e.m. Type A syllables per animal. **H**, Example difference in the distribution (in percent) of pitch (in Hz) in one male for type A syllables before and after M1 lesions.

Lesions of the M1 laryngeal motor cortex in humans or the robust nucleus of the arcopallium (RA) in songbirds severely impair or eliminate the ability to produce learned vocalizations, but do not eliminate the ability to produce innate vocalizations [6], [9], [32]. In contrast, lesions of analogous regions in non-human primates or vocal non-learning birds have been reported to have no effects on the acoustic structure or sequencing of vocalizations [6], [9], [32]–[34]. To test whether mouse ultrasonic songs depend on the motor cortical vocal pathway we discovered, we made chemical ibotenic acid lesions to as much of the laryngeally connected portion of M1 as possible, and performed automated analyses on thousands of song syllables using a custom Matlab program called Syllable Identifier. Our program advances the approach of Holy and Guo [2] to classify 8 common and 3 rare syllable categories (Types A–K) ordered in increasing complexity based the number and direction (downward or upward) of instantaneous pitch jumps separating notes within a syllable (Figure 4A; see methods for more detail). We performed sham surgeries and visual cortex lesions as controls. After recording post-surgical songs, we injected PRV-Bartha into the larynx to verify that layer V projection neurons in M1 were present in the sham treated and visual cortex lesioned animals and eliminated in the M1 lesioned animals (Figure S4A, S4B).

Unlike humans and song-learning birds, we found that mice with bilateral lesions to laryngeally connected M1 still produced what looked and sounded like song (Figure 4F; Audios S2, S3, S4, S5), without any significant change in syllable composition (Figure 4B). However, some spectral aspects of the songs were affected. Qualitative analyses of the songs suggested that relative to both controls (Figure 4C–D) and before the M1 lesion (Figure 4E), mice with M1 lesions had more variation in their syllable frequency modulation (Figure 4F, arrows). Consistent with this finding, quantitative analyses revealed that the M1 lesions caused significant increases in the standard deviation (S.D.) of the pitch distribution of all syllables and frequency modulation (F.M.) within each syllable, without causing a change in the mean frequency (M.F.) of the syllables (Figure 4G; see methods for calculation method). The increased variation in pitch distribution was apparent in plots of individual mice before and after M1 lesions (Figure 4H). Lesion size was not correlated with the song features measured (Simple linear regression; n = 5; Mean Frequency, r^2^ = 0.0543, p>0.5; Standard Deviation of Pitch, r^2^ = 0.0231, p>0.5; Frequency Modulation, r^2^ = 0.4294, p = 0.230; data not shown), but there was very little variation in lesion size (Figure S4C). M1 lesions did not change the amplitude of the songs (Paired Student's t-test; n = 5, p>0.5; data not shown). No significant changes were observed in songs of sham or visual cortex lesion controls (Figure 4D and 4G). These findings indicate that the observed spectral effects were specific to the M1 region. The findings suggest that male mice with a lesion to laryngeally connected M1 have less control over modulating their syllables. When their songs were slowed and pitch-shifted to the human hearing range, M1-lesioned mice sounded less stereotyped from syllable to syllable (compare Audio S2, S3, S4, S5, which correspond to Figure 4C–F).

### Mice require auditory feedback to maintain some features of their songs

The above findings suggest that mice have some neuroanatomical features considered unique to vocal learning species. This could signify that mice are vocal learners or that these features are not truly unique to vocal learners. To test whether mice display a behavioral trait typically associated with vocal learning species we assessed the role of auditory experience and feedback in mouse song behavior. Auditory experience and feedback are necessary during vocal mimicry to guide vocal motor output toward the target sounds in both human speech and birdsong, and to maintain the developed vocalizations, with this requirement being stronger in juveniles than in adults [8]. By contrast, auditory experience and feedback have not been found to be critical for the development or maintenance of normal species-specific songs or calls in vocal non-learning species or of innate calls in vocal learners [5], [10]. For example, humans and song-learning birds show deafened-induced vocal deterioration of acoustic structure in speech and song, but not monkeys and vocal non-learning birds [3], [8], [10], [35], [36].

**Figure 5:**
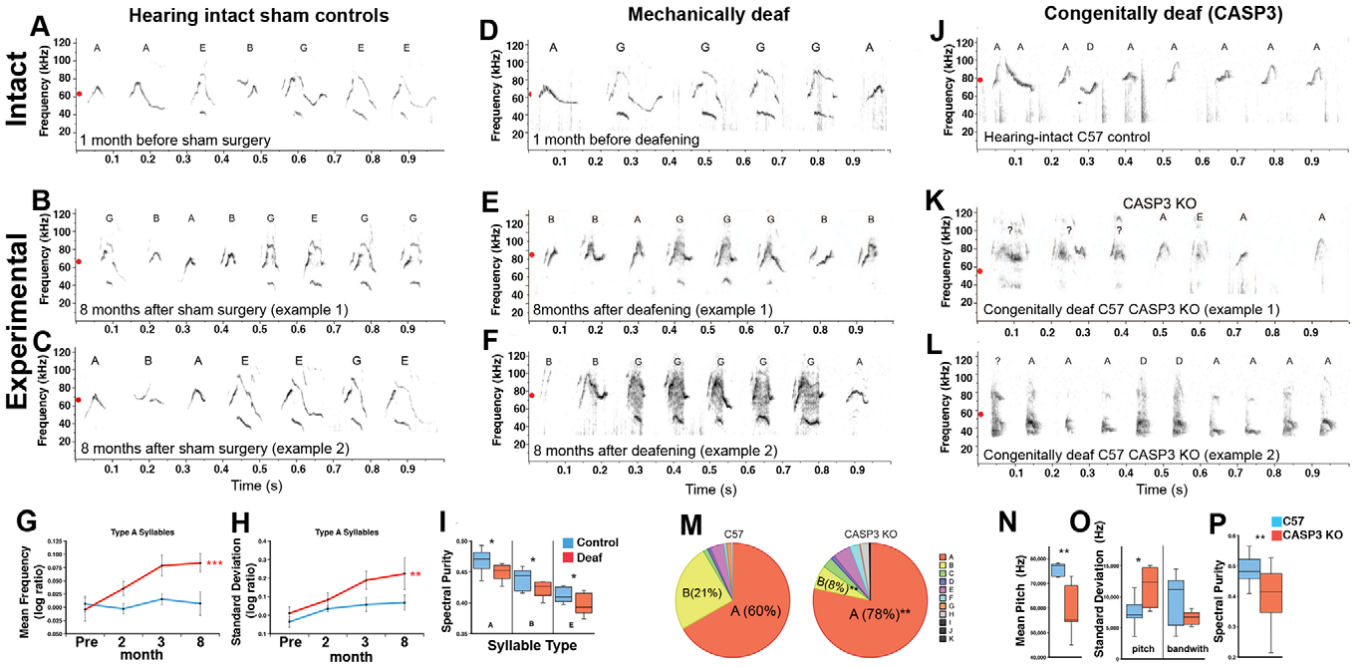
Effects of deafening on mouse song. **A–F**, Sonograms of pre- and post-surgical USVs from hearing-intact and deafened males showing the shift in mean pitch (red dots) and spectral deterioration of post-deafened songs (sonograms correspond to **Audios S6–11**). **G–H**, Mean frequency & standard deviation of the pitch of Type A syllables (expressed as spectral feature scores, SFS, a log-ratio) over 8 post-operative months (** = p<0.01, *** = p<0.001; repeated measures ANOVA with the Bonferroni-Dunn post-hoc test comparing within-group means across recording months; n = 5 per group). Data are plotted as means ± s.e.m. **I**, Box plot of spectral purity of the most common syllable types (Types A, B, and E) in deaf and control groups 8 months after surgery (* = p<0.05; Mann-Whitney U-test between groups; n = 5 per group). Data in G–I are from an average of 3266±536 s.e.m. Type A syllables per animal per month. **J–L**, Sonograms of wild-type B6 and CASP3 KO male USVs (sonograms correspond to **Audios S12–14**). **M,** Pie charts of syllable repertoire composition for B6 and CASP3 KO songs (* = p<0.05; ** = p<0.01; Mann-Whitney U-test; n = 8 B6 and n = 6 CASP3 KO). **N–O**, Box plots of pitch-based features of Type A syllables from the same B6 and CASP3 KO adult males (* = p<0.05; ** = p<0.01; Mann-Whitney U-test). **P**, Box plot of spectral purity of Type A, B, and E syllables combined from B6 and CASP3 KO males. Box plots show the median, 1^st^ and 3^rd^ quartile, and full range. Data in N-P are from an average of 237±109 s.e.m. Type A syllables per animal per group.

To test whether mice require auditory feedback for maintenance of adult acoustic structure, males were deafened at approximately 135 days old by bilateral cochlear removal; age-matched males were sham-operated as controls. Prior to deafening, we allowed the males to have social experience with the opposite sex (overnight exposure to a female) when the males become sexually mature (>35 days old). We found that this typically enhanced their subsequent singing responses to female urine. We then performed at least two months of baseline recordings several times per week to ensure that the spectral features we intended to measure were stable before deafening. Visual inspection of the sonograms post-deafening still revealed recognizable syllables, but with some clear, gradual changes over 8 months that varied in severity from bout to bout. The syllables, especially the more complex Types E–H, were often noisier relative to pre-deafening and sham controls (Figure 5A–F). These songs sounded relatively noisier in pitch-shifted audio recordings (compare Audio S6, S7, S8 to S9, S10, S11). Quantitative analyses of the dominant song syllable category over 8 months (Type A) revealed that the pitch and standard deviation of the pitch across the syllables gradually increased significantly after deafening (Figure 5G, 5H). The changes in the standard deviation of the pitch were similar to the M1-lesioned animals (Figure 4G), but the rate of syllable changes following deafening was much slower than after cortical lesions, taking months rather than days after surgery. Consistent with nosier appearing syllables, Type A and other common syllable types all had significantly lower spectral purity by 8 months (Figure 5I). We wondered if the lower spectral purity could be explained by the deaf mice singing louder and possibly causing microphone distortion, but found that the microphones were not saturated (Figure S5A–B). The acoustic power also did not significantly differ between deaf and hearing-intact groups (Figure S5E). The results also cannot be explained by damage to facial musculature because the sham surgery treated group received the same surgical exposure, and the changes occurred gradually.

To assess the role of auditory experience in mouse song development, we initially attempted to mechanically deafen young pups (<12 days old) but found that the ear canal tissue was too soft for the surgical procedure to work at that young age. We therefore analyzed and compared the songs of normal hearing-intact C57BL/6J (abbreviated B6) males to those of males congenitally deaf due to loss of inner ear hair cells within several days after birth resulting from knockout (KO) of the caspase 3 gene (CASP3) on a B6 background [37]. We found striking differences in the songs of CASP3 KO mice relative to B6 controls (Figure 5H–I). Some of the complex syllables in CASP3 KO songs were highly degraded and barely recognizable, but with some resemblance to normal syllable categories (Figure 5J–L). The simple Type A syllable was produced more often than normal (Figure 5M), had lower mean pitch (Figure 5N), greater standard deviation of the pitch, lower bandwidth (Figure 5O), and lower spectral purity (Figure 5P) consistent with nosier syllables. The difference in mean pitch relative to controls was greater in congenitally deaf versus mechanically deafened mice (17.18 kHz and 3.15 kHz, respectively). Some segments of songs from these deaf mice sounded like squawks and screams rather than whistles when lowered to the human hearing range (compare Audio S12 with S13 and S14). We interpret these vocalizations as songs because they were observed specifically when stimulated to sing with female urine. The syllable degradation was not due to microphone distortion (Figure S5C–D). There was an upward trend in amplitude (loudness) of CASP3 KO songs relative to the B6 controls, but the rise was not significant (Figure S5F). We did not note overt changes in motor behaviors of CASP3 KO animals, suggesting that the changes in pitch might not be attributed to a gross motor deficit. A previous report of mutation of FoxP2 noted changes in the amplitude and pitch of mouse USVs [11], but the effects we obtained in the CASP3 KO animals are the largest that we are aware of for any genetically manipulated animal. Although there is always the possibility of some non-specific effect in genetically manipulated animals, the combined findings of the mechanically deafened and congenitally deaf animals suggest that male mice have some dependence on auditory experience and feedback to develop and maintain some spectral features of their songs.

### Male mice can modify song pitch as a result of social experience

**Figure 6:**
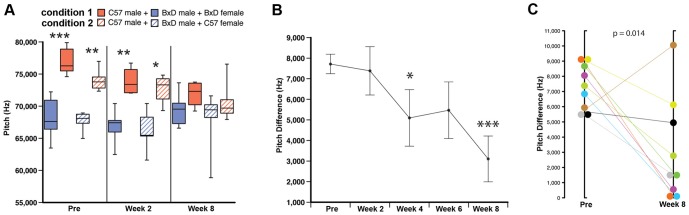
Pitch convergence in B6+BxD male pairs housed with either a B6 or BxD female. **A,** Box plots of Type A syllable pitch from the songs of B6 and BxD males before and over 8 weeks of cross-strain paired housing with either a BxD female (solid boxes) or B6 female (striped boxes) (* = p<0.05; ** = p<0.01; *** = p<0.001; Mann-Whitney U-test; n = 4–7 per time point depending on obtaining a sufficient amount of song). Box plots show the median, 1^st^ and 3^rd^ quartile, and full range. **B,** The mean pitch difference of Type A syllables between the two males in each B6-BxD pair before and over 8 weeks of cross-strain paired housing (* = p<0.05; *** = p<0.001; Student's t-test; Pre: n = 12; Week 2: n = 9; Week 4: n = 6; Week 6: n = 8; Week 8: n = 9; data are plotted as means ± s.e.m.) **C,** Same pre and post data as in (B), but plotted for individual pairs from before (Pre) and at 8 weeks after cross-strain paired housing (p-value reported on the graph; paired Student's t-test; n = 9). Data are from an average of 616±84 s.e.m. Type A syllables per animal per week.

The above experiments indicate that male mice have several neuroanatomical and behavioral features that are necessary for vocal improvisation or imitation, but they do not demonstrate either of these vocal learning abilities. Thus, we asked whether male mice show any evidence of vocal imitation. We noted that two of the strains we studied differed significantly in pitch (C57BL/6J [B6]>B6D2F1/J [BxD]) and this difference (6000–9000 Hz) was reliable in adults housed in acoustically isolated single strain groups (Figure 6A; Pre). We performed an adult social competition experiment by cross-housing a B6 male with a BxD male plus either a B6 (n = 5 pairs) or BxD (n = 7 pairs) female. We surmised that because adult male mice sing to the females as a courtship behavior, introducing a female would induce singing and permit cross-strain acoustic experience. Moreover, if vocal modification were possible, then sexual competition might drive changes to match the song of the strain that the female prefers.

We found that under this competitive social condition, regardless of which female strain was present, the B6 males shifted the pitch of their songs downward over 8 weeks to the range of the BxD males (Figure 6A; pooled data shown in Figure S6). Some BxD males also shifted their pitches slightly upward, but the group stayed within the normal range for their strain (Figure 6A). We wondered if the females could have shaped the male vocalizations instead of males matching the pitch of their male cage mates; however, individual B6 males shifted their pitches closer to the specific BxD male they were housed with, thus reducing the difference between individual pairs over the 8 week period (Figure 6B). Six pairs converged to within 3000 kHz of each other, and of these, 3 pairs were within 0–500 Hz of each other (Figure 6C). All but one pair reduced the difference in pitch by week 8, but this pair had previously achieved a near perfect match (31.81 Hz difference) at 6 weeks before suddenly diverging at 8 weeks (Figure 6C, brown symbol). We also noticed that the B6 males sang on average four times less than the BxD males across all recording sessions (means: B6 = 287±57 syllables, BxD = 1153±142 syllables; s.e.m.: t-test, p<0.001). We could not determine if matching would occur for syllable composition, because even before cross-housing the syllable repertoire percentages of B6 and BxD males did not significantly differ (p = 0.277; Mann-Whitney U-test; n = 7 per group). In summary, our findings indicate that mice can modify and match at least one song spectral feature based on social experience. More experiments are required to determine whether they can match additional song features.

## Discussion

We performed experiments in mice that tested for the presence of five important features traditionally considered to exist as a package unique to vocal learning species: forebrain activation, direct cortical to vocal motor neuron connectivity, forebrain control, auditory feedback, and vocal imitation. We found that mice have these features, although not at the advanced levels found in humans and song-learning birds, but also not completely absent as commonly assumed. We discuss the implications for each of the five features.

### Forebrain activation

Our study is the first that we are aware of to report motor-driven vocalization-related forebrain activation of a laryngeally connected region of primary motor cortex and of the striatum in a non-human mammal. More experiments are required to test if the mouse M1 layer V and striatal neurons exhibit pre-motor activity during USV song production. While our study on mice was in preparation, several studies challenged the claim that non-human primates do not have cortical regions active in the production of vocalizations, using PET neuroimaging, IEG mapping, and electrophysiology [38]–[41]. The PET study conducted in chimpanzees found that a brain region in a similar location as Broca's area (i.e. ventral prefrontal cortex) was activated during vocalizing [38]. The IEG studies conducted in marmosets found induced gene expression in prefrontal cortex after animals vocalized [39], [40]. The electrophysiology study conducted in macaques found neural firing in the ventral premotor cortex during vocalization [41]. However, the studies did not report whether there was differential activation of M1 or striatum. Additionally, these studies did not control for potential activation driven by auditory feedback by reducing or eliminating auditory input. In the electrophysiology study, neurons fired when the monkey's produced conditioned vocalizations but not when they produced similar vocalizations spontaneously, suggesting that the brain region is not responsible for motor programming of the vocalizations. If the non-human primate results can be extended to eliminate the possibility of auditory feedback and show some control over the spectral structure of vocalizations, as the present study showed in mice, then such a finding would indicate that forebrain activation for some spectral modulation of vocalizations could be a common feature in mammals.

### Connectivity

A direct projection from mouse M1 to Amb was our most unexpected finding, considering prior claims over the past 50 years of its absence in vocal non-learning species and its importance for the evolution of vocal learning and speech [1], [6], [7], [9], [13], [14], [18], [19], [24]–[26] (Text S1). It is possible that the projection we found is not functional. However, our M1 lesion results suggest that the projection is necessary to keep mouse song stereotyped. A hypothesis of a non-functional projection would also not explain the difference we found between mice and so-called vocal non-learning species with claims of no axons from the cortex. An alternative possibility is that a direct projection was missed in non-human primates and other species. Most publications on non-human primates over the past 40 years show drawings of the brainstem at the level of Amb but not the primary data [9], [20], [22]. Thus, we obtained brain sections from the authors of one of the most recent studies [22], and verified that the sections lack BDA-labeled axons from the ventral premotor cortex (Area 6V) in Amb and contain labeled axons in the reticular formation dorsal to Amb (Figure S7). Area 6V in non-human primates when stimulated produces laryngeal muscle deflection and makes only an indirect projection to Amb through the reticular formation [9], [14] (Figure 1E). Area 6V is in the premotor cortex, whereas the projection we found in mice is from M1 rather than premotor M2. To explain the differences among species (with the belief that mice and most other non-primate mammals do not have a laryngeal M1), Simonyan and Horwitz [14] proposed that the evolution from innate to learned vocalizations may have involved first a relatively unique appearance of a laryngeal motor cortex in Area 6V in a non-human primate ancestor that then later shifted in location and function to M1 in humans, simultaneously forming a direct projection to Amb.

If our alternative explanation is correct, then the direct projection in non-human mammals could be much sparser than in vocal learning birds [13] and sparser than our visual inspection of the available primary data in humans [19], making it difficult to find using standard tracing techniques. In fact, although prior tracer studies have claimed that cats, rats, tree shrews, squirrel monkeys, macaques, and pigeons all “lack" a laryngeal M1 (or syringeal arcopallium for birds) with axons that project to vocal motor nuclei (mammalian Amb; avian XIIts; even with injections larger than those we placed in mice) [9], [13], [22], the very first study using a neural degeneration technique in chimpanzee and macaque did state (but not show) that after M1 lesions: *“Only very few, if any, degenerating elements were found among the cells of the ambiguus nuclei."* [20]. This suggests the possibility of a sparse projection that may have not been followed up on.

In our mice studies, we used a transynaptic tracer to identify laryngeally connected motor cortex, whereas the previous studies used conventional tracers starting from orofacial cortical areas or premotor Area 6V. Therefore, the correct cortical area may not have been injected, or a sparse projection may not have been easily noticed. Moreover, it is nearly impossible to inject a tracer exclusively in Amb in mammals or XIIts in birds without leakage in the surrounding reticular formation, due to the motor nucleus' small diameter. Partly consistent with our alternative hypothesis, a recent study in rats using the same PRV-Bartha transynaptic tracer injected in the larynx found a few isolated labeled cells in M1 more than 120 hrs after injection into laryngeal muscles [30]; however, that study did not discuss the possible implications of this finding or test for a direct projection. Thus, if a transynaptic tracer approach were to reveal a M1 region that projects directly to Amb in non-human primates and other species, even sparsely, then such a finding would prompt a serious re-evaluation of the hypothesis that the direct projection is a specialization of vocal learners [1], [6], [7], [9], [13], [14], [18], [19], [24]–[26] (Text S1). Confirmed absence would suggest real neuroanatomical differences between species, and that mice have connectivity closer to humans and song-learning birds than they do to non-human primates.

### Forebrain control

Area 6V is not required for producing non-human primate vocalizations, and it remains to be determined whether it modulates vocalizations or other laryngeal functions for voluntary control of breathing and eating [9], [33]. In contrast, we find in mice that although the singing-activated, laryngeal connected M1 region is also not necessary for generating song, it is necessary for modulating some acoustic features of song. The differences in the songs before and after M1 lesions appear to be the reverse of developmental changes made in the transition from juvenile to adult mouse vocalizations [42]. That is, M1 lesions shift the pitch distribution to a variable and more juvenile-like state. One possible interpretation of these findings is that the mouse laryngeal M1 region exerts fine acoustic pitch and frequency modulation control of brainstem-generated innate vocalizations. It is also possible that M1 controls respiration during vocalization. However, this is not a distinguishing feature between vocal learners and non-learners, because the RA nucleus of songbirds controls both respiratory premotor and vocal motor neurons [13]. Changes in respiration or supralaryngeal filtering would also not be expected to affect the pitch or standard deviation of the pitch distribution [43]. Moreover, even if the observed effects were due to a respiratory mechanism, similar results have not been reported after M1 lesions in non-human primates [9], [33]. Our interpretation is that mouse USV song syllables may be more similar to male zebra finch long calls, which contain both cortical learned and brainstem generated innate components. Lesions of RA in zebra finches eliminate the learned features of calls leaving a basic innate template generated by the brainstem [32]. Our findings also suggest that a more rigorous analysis should be conducted on other species to determine if subtle effects of cortical lesions were missed.

### Auditory Feedback

Although auditory feedback was necessary for production of normal acoustic features of mouse song, the deafening-induced deficits in adults were less dramatic than those reported in humans and song-learning birds [8], [44], [45], indicating more of an innate component to the mouse song syllables. However, deafening-induced deterioration is also less dramatic and takes months to develop for learned contact calls in the budgerigar, a small parrot [44]. It is possible that other factors could have affected the vocalizations of deaf mice, such as potential hormonal changes linked to altered social experience from being deaf. Even if non-auditory factors are at work, they apparently do not similarly affect the spectral properties (pitch, frequency modulation, spectral purity) of vocalizations in deafened chickens, suboscine songbirds, cats, or non-human primates [3], [10], [26], [34], [36], [46]. That is, to the best of our knowledge, such spectral effects (even subtle ones) have not been reported in vocal non-learning birds or non-human primates [3], [9], [10], [35]. However, some acoustic features such as duration and loudness can be affected in these species [3], [10], [34], [36], [46], most likely resulting from the brainstem-controlled Lombard effect [33], [47]. Yet even subtle deafening-induced and developmentally regulated spectral changes to birdsong require forebrain vocal circuits [45], [48]. Therefore, our findings suggest either real species differences exist between mice and supposed vocal non-learners or that a more fine-grained re-analysis of auditory feedback dependence in vocal non-learners is in order.

While our paper was under review, another study published findings on the vocalizations of congenitally deaf mice from knockout of the otoferlin gene on a mixed 129 ola/C57N background [49]. Like our study, they did not find a significant difference in amplitude between deaf and control animals; however, unlike our study they claimed to find no other differences in the syllables of the congenitally deaf versus hearing-intact animals. Relative to our approach, they used a much simpler classification scheme (2–3 syllable types) for all of their non-amplitude analyses that mixes syllables with differences in acoustic features that can be larger than the differences we find between hearing-intact and deaf animals. They also did not analyze the spectral features that we report as different (mean pitch, spectral purity, frequency variance, standard deviation of the pitch distribution). Nevertheless the sonograms presented appear not to show differences as large as those in some of the sonograms we show, particularly for the CASP3 KO animals. The combined findings suggest that the differences between studies could be methodological and/or biological.

### Vocal Imitation

Our finding that housing two male mice with a female can result in pitch convergence between those males indicates that mice could possibly “learn" at least one acoustic feature in their vocalizations. The drops in B6 pitch were large (4470±86 Hz s.e.m.) and of similar magnitude to changes used as evidence for vocal learning in bats [50]. Although pitch convergence has been reported in non-human primates, the changes were not reliable, and divergence from the normal range occurred in only one of eight animals [51]. That males in our experiment matched the pitch of their specific cage mates suggests that the effects likely occurred through auditory experience with each other. An alternative explanation is that the decline in B6 is due to age related hearing loss in this strain. However, we believe this explanation is unlikely for the following reasons: these mice exhibit moderate hearing loss later in life (after 6 months) [52], and we performed the experiments when they were less than 5 months old; the animals converged to the specific pitch of their cage mates; there was 4 week age offset between the mice in the two treatments studied, with similar results; and, we did not see a similar pitch decrease in one month of recording before cross housing. It is also possible that females shaped the songs by non-vocal reinforcement, as reported for some songbirds [53]. Another possibility is that because most B6 males shifted in the same direction, were smaller than the BxD males, and sang less than BxD males, the B6 males may be matching the pitch of the dominant male whenever a female is present. It is still unclear if mice can match other song features (i.e. frequency modulation and syllable sequencing), if supposed vocal non-learners are capable of pitch convergence, and whether mice (and other supposed vocal non-learners) use similar forebrain circuits as known vocal learners to achieve this feat. This can now be tested in mice by lesioning or blocking M1 and assessing pitch convergence.

A related report published while our paper was being prepared claimed that two strains of mice that sing very different songs (C57BL/6 [B6] & BALB/c) are not able to imitate each other, including their pitch [54]. Several issues need to be addressed to resolve the differences with our study. First, the mice in that study were ‘tutored’ for a short period (3 weeks) very early in development, which was not long enough to obtain pitch matching in our studies. Second, the ear canal is closed for almost two weeks at the beginning of this period. Third, after cross-fostering but prior to testing, the tutored mice were placed in mixed strain housing in an acoustically unshielded colony for a much longer period (7 to 17 weeks) than the cross-fostering phase. In effect, the tutored mice had the opportunity for significantly more auditory experience with the songs of their own strain before testing. Given the demonstrated predisposition of some vocal learning species to learn their own species-typical songs [8], there is a possibility that the previously tutored mice actively selected songs of their own strain while in the colony. Fourth, the auditory environment was not monitored to confirm that the tutor mice sang. Our findings indicate that the social conditions and the amount of time cross-housed can significantly influence the results obtained.

In summary, based on our findings and the body of literature on animal vocal communication, we propose that vocal learning and the associated traits may not be dichotomous as commonly assumed, but instead it may fall into multiple distinct categories along a continuum, with vocal mimics and some supposed vocal non-learning species at either extreme. One category could be defined by the direct cortical projection to vocal motor neurons, where some species have it and some do not; for those that have it, the strength and density of the projection could be positively correlated with the degree of limited to advanced vocal learning. Other categories could be defined by the type of auditory input into vocal motor pathways, which would influence the level of dependence on auditory feedback to maintain vocal motor output. Given the currently available published data, mice would appear intermediate to species like chickens/monkeys and songbirds/humans on such a continuum. A prediction of this hypothesis is that vocal learning would be found among different species to various degrees, an idea supported by a recent claim for goats [55], which we interpret as having limited vocal learning abilities compared to humans and song-learning birds. An alternative interpretation of our results is that mice are vocal non-learners under a dichotomous classification scheme. This conclusion would require a reappraisal of what defines vocal learning and the associated traits. It would require that the presence of a primary motor cortical region with a direct projection to laryngeal motor neurons, dependence on auditory feedback to develop and maintain acoustic structure of vocalizations, and the ability for pitch matching not be sufficient for classification as a vocal learner. Falsifying either of these hypotheses will require more detailed study on mice and other species. For a more detailed discussion of these alternatives, see Arriaga and Jarvis [4]. At a minimum, our findings suggest that male mice could be useful as a genetically tractable model to study some of the vocal communication traits long thought unique to humans and other complex vocal learners. Our results also identify parts of a vocalization brain system for the many investigations that use mice as models to study neural mechanisms of vocal communication in the context of neuropsychiatric and neurodegenerative disorders, including autism, Parkinson's disease, Tourett's syndrome, motor control of the larynx, spasmodic dysphonia, and social disorders [1], [4], [56].

## Materials and Methods

### Animals and Song Recordings

Adult males and females of the B6D2F1/J (BxD), C57BL/6J (B6), B6.129S1-Casp3^tm1Flv^/J (CASP3), and BALB/c strains were purchased from the Jackson Laboratory (Bar Harbor, Maine). All mice were group housed and kept on a 12 hr light/dark cycle. At 5 weeks old, males were socialized by spending at least one night with an adult female. We selected males for behavioral experiments that readily sang in response to female urine. Therefore, the probability that the males in each of the groups in our experiments were from the same litter is very low. For recordings, males were placed into a recording box with fresh bedding, allowed to acclimate overnight for IEG experiments or 15 min for behavior only experiments, then stimulated to sing by presenting 200 µL of female urine directly into the bedding. Sounds were recorded with UltraSoundGate CM16/CMPA ultrasound microphones that feature a flat frequency response from 30–130 kHz and an UltraSoundGate 416–200 recording interface (Avisoft Bioacoustics). The microphones were suspended over the center of the recording box to minimize the differences in sound pressure level reaching the microphone due to varying horizontal orientation. Sounds were digitized at 250 kHz, 8 bits and captured to disk as .WAV files using Avisoft Recorder USG (Avisoft BioAcoustics). Example songs were pitched and slowed down to the human hearing range using Raven 4.0 (Cornell Laboratory of Bioacoustics). For playback studies, a set of recorded songs from the singing males of the IEG study were played at normal pitch, through an Avisoft ultrasound amplifier Model #70101 and speaker Model #60401 (Avisoft Bioacoustics). The same songs were used for all playback sessions, and sessions were recorded to ensure that no USVs were produced by the listening mouse. Mice that responded with USVs were not included in the analysis. All recordings and playbacks were conducted in dark, enclosed isolation chambers. Movement in the dark was recorded with a Speco Technologies VL-62 color infrared camera, the video was saved to tape, then digitized on a Canopus ADVC110 analog-to-digital converter at full resolution, and stored to disk as MPEG video files.

### Behavioral Molecular Mapping

Adult male BxD mice were acclimated by placing them in a dark 15″×24″×12″ sound-attenuating recording chamber overnight. The following day, after a period of 3 hrs with little movement and no ultrasonic songs, males were presented with an olfactory or auditory stimulus. Five normal males (Singing & Hearing group) and 5 deafened males (Deaf-Singing group) were stimulated to sing by presentation of 0.1 cc of fresh urine from BALB/c females, pippetted on the bedding through a small covered opening on the top of the sound chamber. Five males were stimulated to explore the home cage without singing by presentation of 0.1 cc of 10% EtOH (Non-Singing group) to control for possible olfaction and movement-induced IEG expression in the singing groups. Olfactory stimuli were presented at 5 min intervals throughout the 30 min recording session to maintain exploratory and vocal behavior. We had one additional male that spontaneously sang without urine stimulation and showed the same IEG pattern as the urine stimulated singing animals (not shown). Five males were stimulated with 30 min of continuous presentation of identical USVs (Hearing Only group) recorded from a normal adult male played through an Avisoft ultrasound amplifier and speaker as described above. All other procedures were as described in the main text.

### *In Situ* Hybridization

Immediately after the 30 min behavioral sessions, animals were sacrificed by decapitation without anesthesia, as the IEG changes can be sensitive to manipulation within 5–10 minutes of handling. Brains were removed, embedded in Tissue-Tek (Sakura Finetek), frozen on dry ice and then stored at −80°C. Coronal 12 µm sections were cut through the entire brain on a cryostat and every other section was mounted on silanated slides in series of 10. Frozen sections were processed for *in situ* hybridization with a ^35^S radioactively labeled riboprobe made from cDNAs for mouse egr-1 and rat arc, and processed for emulsion autoradiography following a previously described protocol [57]. The egr-1 probe was generated from PCR-amplified sequences of the pCMV-Sport6-egr-1 plasmid containing the full-length mouse egr-1 cDNA (3.1 kb) insert from our own library (Pioneer Clone F6). The arc probe was generated from PCR-amplified samples of a 1.5 kb sequence of the rat arc cDNA, prepared according to a previously described protocol [57].

### Deafening

Adult male mice 77 to 87 days old were deafened by bilateral cochlear removal. Anesthesia was induced with 5% isofluorane in oxygen and maintained by intramuscular injection of ketamine-xylazine (75 mg/kg ketamine; 5 mg/kg xylazine). A retro-aural incision was made and the skin and muscle were retracted to reveal the tympanic bulla. The lateral wall of the bulla was punctured to reveal the cochlea, and a pair of fine forceps was used to remove the tympanic membrane, stapes and parts of the cochlear walls until no cochlear structure was visible. Sham surgery treated animals received all of the same treatments, except the bulla was not punctured and the tympanic membrane was not accessed.

### Behavioral Analysis

Acoustic waveforms were processed using custom MATLAB programs that we modified from code written by Timothy E. Holy (Washington University) [2] and that we called Syllable Identifier, made available upon request. A sonogram was computed from each waveform (256 samples/block, half-overlap), thresholded to eliminate the white noise component of the signal, and frequencies outside 35–125 kHz were truncated. Syllables with duration longer than 10 ms were identified and classified by presence or absence of instantaneous ‘pitch jumps’ separating notes within a syllable. The morphologically simplest note type doesn't contain any pitch jumps (Type A). The next most complex were those containing two notes separated by a single upward or downward pitch jump (Types B & C, respectively). More complex syllables were identified by the series of upward and downward pitch jumps occurring as the fundamental frequency varies between notes of higher and lower pitch (Types D–K). Much rarer syllable types (<1%) were grouped as other.

The following spectral features were calculated from the sonograms of each of the classified syllables types: Standard deviation of pitch distribution, mean frequency, frequency modulation, and spectral purity. Frequency modulation was measured as the frequency variance, or the squared deviation of peak frequencies from the mean peak frequency, averaged over the length of the syllable. Spectral purity was calculated as the instantaneous maximum power at the peak frequency normalized by the instantaneous total power in the spectrum, averaged across the entire syllable; a pure tone would have a spectral purity of 1, and white noise would approach 0. We also calculated starting frequency, ¼ frequency, ¾ frequency, final frequency, minimum frequency. From these measures, the mean value for each spectral feature (FV) was calculated for each recording epoch (single session, weeks or months depending on the experimental design) for all syllable types. For longitudinal data, we took the logarithm of the normalized mean value for each epoch (n) as the spectral feature score (SFS) such that: SFS(n) = log10[FV(n)/FV(1)]. This log ratio allowed us to obtain a relative difference to the pre-treatment conditions across animals, which could differ in their absolute values. Essentially, the SFS gave us a measure of the change in each measured acoustic feature for each animal normalized to their own pre-treatment baseline. The log ratio made it symmetrical around zero. This approach allowed us to easily visualize and compare both decreases and increases in individual acoustic features across scales and across animals.

Digitized videos were coded for periods during which the animal was sitting still or moving throughout the home cage using the behavioral coding software Annotation by SaySoSoft (v1.0, http://www.saysosoft.com/). The durations of all locomotor and rotational movement recorded were summed to determine the total amount of movement produced by each animal.

### Gene Expression Analysis

Photomicrographs were taken from autoradiographs of hybridized sections. Regions of interest (ROIs) were defined in three serial sections for each brain area on inverted images in ImageJ (NIH, Bethesda). The mean pixel value was recorded for each ROI (Mroi), three regions of each glass slide with no brain tissue (Mbkgnd), and control areas with no difference in the background-adjusted mean pixel values across groups (Mctrl). The control areas with no difference were: 1) the ventral striatum for cingulate cortex, motor cortex, somatosensory cortex, and anterodorsal striatum (Figure 2B); 2) the midbrain reticular nucleus for the auditory cortex (Figure 1D). These values were used to calculate the expression score (ES) for each ROI as follows: ESroi = log10[(Mroi−Mbkgnd)/(Mctrl−Mbkgnd)]. The values were log transformed to visualize comparable magnitudes for expression differences above and below silent control levels in the experimental animals.

### Tracer experiments

For retrograde tracing from laryngeal muscles, we used a recombinant strain of Psuedorabies Bartha (PRV-Bartha) expressing enhanced Green Fluorescent Protein (eGFP) under the control of the histomegalovirus immediate early gene promoter [27], [28]. Live virus was received from the laboratory of Dr. Lynn Enquist at Princeton University at a titer of 1×10^9^ pfu/mL, aliquoted at 4 µL per tube, then stored at −80°C, and thawed immediately before injection. General anesthesia was induced with 1% isofluorane and maintained by intramuscular injection of ketamine-xylazine (75 mg/kg ketamine; 5 mg/kg xylazine). A midline incision was made from the sternum to the hyoid bone. The portion of the sternohyoid muscle covering the larynx was removed. Five 200 nL injections were made 1 min apart into the cricothyroid laryngeal muscle using a Nanofil microsyringe system with a 34 gauge stainless steel needle (World Precision Instruments, Sarasota, FL). After 5 min, the microinjection pipette was retracted, and the injection was repeated for the cricoarytenoid lateralis muscle. A single break in the fascia was made for each muscle and sealed with TissueMend adhesive (Veterinary Products Laboratories) to prevent spread of virus to other tissues.

For anterograde tracing of cortico-bulbar projections to nucleus ambiguus we injected 7.5% BDA (Biotinylated Dextran Amine, 10000 MW; Sigma) in sterile water into the motor cortex of 6 adult male mice (5 bilateral and 1 unilateral). Following induction of anesthesia, as above, the scalp was retracted and a small craniotomy made over the injection site. Injections of BDA were made through a glass micropipette using the Nanoject II microinjector (Drummond Scientific) at 4 sites 0.550 mm from the brain surface (50–90 nL per site) along a track 1.2 mm lateral and −0.2 to 0.4 mm anterior to Bregma. Then, 6–11 days later, three of these mice were injected with 0.5 µL of 1% CTb (Cholera Toxin Subunit b) in sterile water in the two laryngeal muscles, as described above for PRV-Bartha. Two days after CTb injections, the mice were sacrificed and transcardially perfused as described below.

### Chemical Lesions

For chemical lesions of cortex we injected 7.5% ibotenic acid in sterile water bilaterally (220 nL per injection site) into the motor cortex of 6 adult mice, and into the visual cortex bilaterally (220 nL per injection site) of 4 adult mice. The coordinates for the motor cortex were as above for the tracer experiments, and for visual cortex were 3 mm caudal and 1.5 mm lateral to Bregma. 5 adult mice received sham surgeries in which the scalp and skull were opened, as described above, but no injection was made. After recording USVs three weeks after surgery, all mice were injected with PRV-Bartha in the laryngeal muscles, as described above. To quantify the lesions we counted the number of surviving PRV-Bartha-labeled layer V pyramidal cells in M1 in 7 serial sections per hemisphere. Lesion sizes were expressed as a percentage of cells eliminated from a baseline of 102±16 cells (s.e.m.) counted from 7 similarly quantified unlesioned hemispheres. When calculating the spectral feature scores for post-operative songs one lesion case was confirmed to be an outlier on three univariate feature scores using the Dixon Q Test (Q_90%_ = 0.560, Q_95%_ = 0.625; Bandwidth: Q = 0.5852; Range: Q = 0.5727; Frequency Variance: Q = 0.7244) and the multivariate Mahalanobis Jackknifed Distance using 11 spectral feature scores (Chi Square (97.5%) JMD = 4.6819; Outlier: MJD = 7.9143). This case was excluded from further analysis.

### Immunohistochemistry for tracers

Unless otherwise noted washes of brain sections were 3 times for 5 min in 0.1 M PBS. Animals were given an overdose of pentobarbital sodium and perfused transcardially with 0.9% saline followed by 4% paraformaldehyde in 0.1 M PBS. Brains were removed, post-fixed in 4% paraformaldehyde overnight, and cryoprotected in 30% sucrose in PB until sectioned. 40 µm coronal sections were cut on a cryostat into 0.1 M PBS.

For visualizing BDA, free-floating sections were quenched 30 min in 0.3% H_2_O_2_, then reacted 1 hr in ABC solution (VECTASTAIN Elite Kit, Vector Labs). Sections were then washed 3 times for 10 min in PB, and developed for 15 min in 0.05% 3,3′-diaminobenzidine (DAB) solution (Sigma, #D5905) with nickel (DAB Substrate Kit, Vector Labs) to give a black reaction product.

For double labeling with CTb, following BDA detection, free-floating sections were blocked 1 hr in 0.3% PBST with NRS (VECTASTAIN Elite Kit, Vector Labs). Blocked sections were incubated 2 hrs at RT in goat anti-CTb (1∶10000 dilution, List Biological Laboratories) followed by 1 hr in rabbit anti-goat biotinylated secondary antibody (VECTASTAIN Elite Kit) and then reacted 30 min in ABC solution. DAB staining was as above, but for 3 min without nickel to give a brown reaction product.

For labeling of eGFP expressed from the PRV-Bartha recombinant vector, free-floating sections were quenched as above, then blocked 30 min in 0.3% PBST with NGS (VECTASTAIN Elite Kit). Blocked sections were reacted for 3.5 hrs at RT in rabbit anti-eGFP (1∶1000 dilution, Open Biosystems), followed by 1 hr incubation in goat anti-rabbit biotinylated secondary antibody (VECTASTAIN Elite Kit) or 2 hrs at RT in donkey anti-rabbit secondary antibody conjugated to Alexa-fluor 488 (1∶500 dilution, Invitrogen). ABC reaction and DAB staining were the same as for CTb detection, but for 8 min.

## Supporting Information

Audio S1 **Example of a normal adult BxD mouse song (audio corresponds to sonogram of USVs in** **Figure 1A** **).**
(WAV)

Audio S2 **Example of adult BxD mouse song 1 week before sham brain lesion surgery (audio corresponds to sonogram of USVs in** **Figure 4C** **).**
(WAV)

Audio S3 **Example of adult BxD mouse song 3 weeks after sham brain lesion surgery (same mouse as Audio S2; audio corresponds to sonogram of USVs in** **Figure 4D** **).**
(WAV)

Audio S4 **Example of adult BxD mouse song 1 week before lesions in laryngeally connected M1 (audio corresponds to sonogram of USVs in** **Figure 4E** **).**
(WAV)

Audio S5 **Example of adult BxD mouse song 3 weeks after lesions in laryngeally connected M1 (same mouse as Audio S4; audio corresponds to sonogram of USVs in** **Figure 4F** **).**
(WAV)

Audio S6 **Example of adult BxD mouse song 1 month before sham deafening surgery (audio corresponds to sonogram of USVs in** **Figure 5A** **).**
(WAV)

Audio S7 **Example of adult BxD mouse song 8 months after sham deafening surgery (same mouse as Audio S6; audio corresponds to sonograms of USVs in** **Figure 5B** **).**
(WAV)

Audio S8 **Example of adult BxD mouse song 8 months after sham deafening surgery (same mouse as Audio S6; audio corresponds to sonograms of USVs in** **Figure 5C** **).**
(WAV)

Audio S9 **Example of adult BxD mouse song 1 month before deafening by cochlear removal (audio corresponds to sonogram of USVs in** **Figure 5D** **).**
(WAV)

Audio S10 **Example of adult BxD mouse song 8 months after deafening by cochlear removal (same mouse as Audio S8; audio corresponds to sonograms of USVs in** **Figure 5E** **).**
(WAV)

Audio S11 **Example of adult BxD mouse song 8 months after deafening by cochlear removal (same mouse as Audio S8; audio corresponds to sonograms of USVs in** **Figure 5F** **).**
(WAV)

Audio S12 **Example of normal adult C57 mouse song (audio corresponds to sonograms of USVs in** **Figure 5J** **).**
(WAV)

Audio S13 **Example of congenitally deaf CASP3 KO mouse song (audio corresponds to sonogram of USVs in** **Figure 5K** **).**
(WAV)

Audio S14 **Example of congenitally deaf CASP3 KO mouse song (audio corresponds to sonogram of USVs in** **Figure 5L** **).**
(WAV)

Figure S1 **Behavioral-molecular mapping of mouse song system forebrain areas with arc and expression of IEGs in control areas.** **A–D**, Dark-field images of cresyl violet stained (red) coronal brain sections showing singing-induced arc expression (white) in the Hearing & Singing male mice, and reduced expression in the A1 cortex of Deaf-Singing male mice. Sections are adjacent to the same animals shown in Figure 2A–B, D–E. Scale bars, 1 mm. **E–F,** Raw expression measurements of arc and egr-1 mRNA in the ventral striatum (**E**) and midbrain reticular (Rt) nucleus (**F**) showing no difference among the four groups (Kruskal-Wallis H-Test; n = 5 per group; ventral striatum, egr-1: p = 0.3, arc: p>0.5; midbrain reticular nucleus, egr-1: p>0.5, arc: p = 0.070; data are plotted as means ± s.e.m.). These brain areas were used to normalize expression in other brain regions (see methods). Abbreviations are as in Figure 2.
(TIF)

Figure S2 **Amount of movement and IEG expression levels in singing active and laryngeal connected M1+M2 region.** Shown are linear regressions of arc (**A**) and egr-1 (**B**) expression scores (y-axis) relative to the total time spent moving in the cage (x-axis) during the recording session. Movement was scored with the program Annotation by SaySoSoft, and the total time spent making ambulatory back and forth and rotational movement calculated (see methods). Even though there were large differences among some animals, such as two mice in the Hearing Only group that remained relatively still, there was no correlation between the amount of movement and the amount of IEG expression.
(TIF)

Figure S3 **M1 axons in the brainstem.** **A**, Low power view of a coronal brainstem section containing CTb-labeled motor neurons in Amb (brown) from an injection in laryngeal muscles and M1 axons (black) from an injection of BDA into M1 (similar plane of section as in Figure 3A). Only BDA label axons can be seen in the cortico-pyramidal (Pyr) track at this low magnification. Abbreviations: Amb, nucleus ambiguus; Pyr, pyramids; mRF, reticular formation directly medial to Amb; dRF, reticular formation dorsal to Amb. **B**, High magnification of BDA labeled axon (black) from M1 in Amb that splits near a CTb labeled motor neuron cell body (brown), with one axon branch making a large bouton-like contact (arrow) and the other branch wrapping around the cell body (arrow heads). **C**, M1 axons (black) running along and near a large Amb motor neuron dendrite that radiates out from Amb. **D,** Axons (black) in a localized region of the reticular formation directly medial to Amb, where ambiguus motor neuron dendrites pass nearby (brown). **E,** No axons were seen in the reticular formation further medial and dorsal to Amb. Greyish dots without labeled axons are artifacts of the double labeling protocol. Scale bars: 1 mm for a; 10 µm for B–E.
(TIF)

Figure S4 **Verification and quantification of M1 lesions.** **A**, eGFP labeled Layer 5 M1 neurons from a PRV-Bartha-eGFP tracer injected in laryngeal muscles of a sham control animal; this result replicates the findings shown in Figure 3C–D, bringing the total number of animals with such backfilled cells to 19. **B**, Elimination of PRV-Bartha back-traced premotor neurons in M1 following chemical lesions. Scale bars, 1 mm. **C,** Distribution of lesion sizes based on elimination of PRV-Bartha-eGFP labeled layer 5 pyramidal cells in M1 lesioned animals (12 cerebral hemispheres in 6 mice) relative to an average of sham controls (n = 5 mice). Most lesions eliminated more than 85% of traceable neurons, with a mean lesion size of 94% (red dot).
(TIF)

Figure S5 **Amplitude of songs from deafened male mice.** **A–B,** Waveforms of song excerpts used to generate the sonograms in **Figure 5B and 5F** of a hearing-intact sham control and a deafened adult male, respectively. **C–D,** Waveforms of song excerpts used to generate the sonograms in **Figure 5H and 5I** **,** of a wild type C57 and a congentially deaf CASP3 KO male, respectively. The microphones were not saturated during these recordings; saturation causes clipping at the upper and lower ends of the waveforms. **E**, Normalized amplitudes (SFS) show no differences in sham-operated and deaf adult male mice before and 8 months after surgery (Two-way repeated-measures ANOVA; Treatment: F = 0.203, p>0.5; Recording Session: F = 2.698, p = 0.139; Treatment×Recording Session: F = 0.038; p>0.5; n = 5 per group). **F**, Normalized amplitude (SFS) show a trend of increased amplitude but the difference is not significant in adult CASP3 KO versus C57 male mice (Student's t-test; p = 0.147; n = 8 C57 and n = 6 CASP3 KO).
(TIF)

Figure S6 **Pooled data for pitch convergence in C57/BxD male pairs housed with either a C57 or BxD female.** Group mean pitch of Type A syllables from the songs of C57 and BxD males before and over 8 weeks of cross-strain paired housing, pooled across female strain (BxD female or C57 female). Pitch convergence was also found in the pooled data (* = p<0.05; ** = p<0.01; *** = p<0.001; Student's t-test; Pre: n = 12 C57, n = 12 BxD; Week 2: n = 8 C57, n = 12 BxD; Week 4: n = 6 C57, n = 11 BxD; Week 6: n = 8 C57, n = 11 BxD; Week 8: n = 9 C57, n = 12 BxD). Box plots show the median, 1^st^ and 3^rd^ quartile, and full range.
(TIF)

Figure S7 **Anterograde tracing from Area 6V in rhesus monkeys.** **A**, BDA labeled axons from Area 6V present in the reticular formation dorsal to nucleus ambiguus. **B**, Lack of axons in nucleus ambiguus where the motor neurons (MN) are located. Sections are from Kristina Simonyan, and were used for the drawings in a previous study [12].
(TIF)

Text S1 (DOCX)
