# Supplementary figures and images for: Of Mice, Birds, and Men: The Mouse Ultrasonic Song System Has Some Features Similar to Humans and Song-Learning Birds
Source: PLoS One. 2012 Oct 10;7(10):e46610. doi: 10.1371/journal.pone.0046610 (PMC3468587; doi:10.1371/journal.pone.0046610)

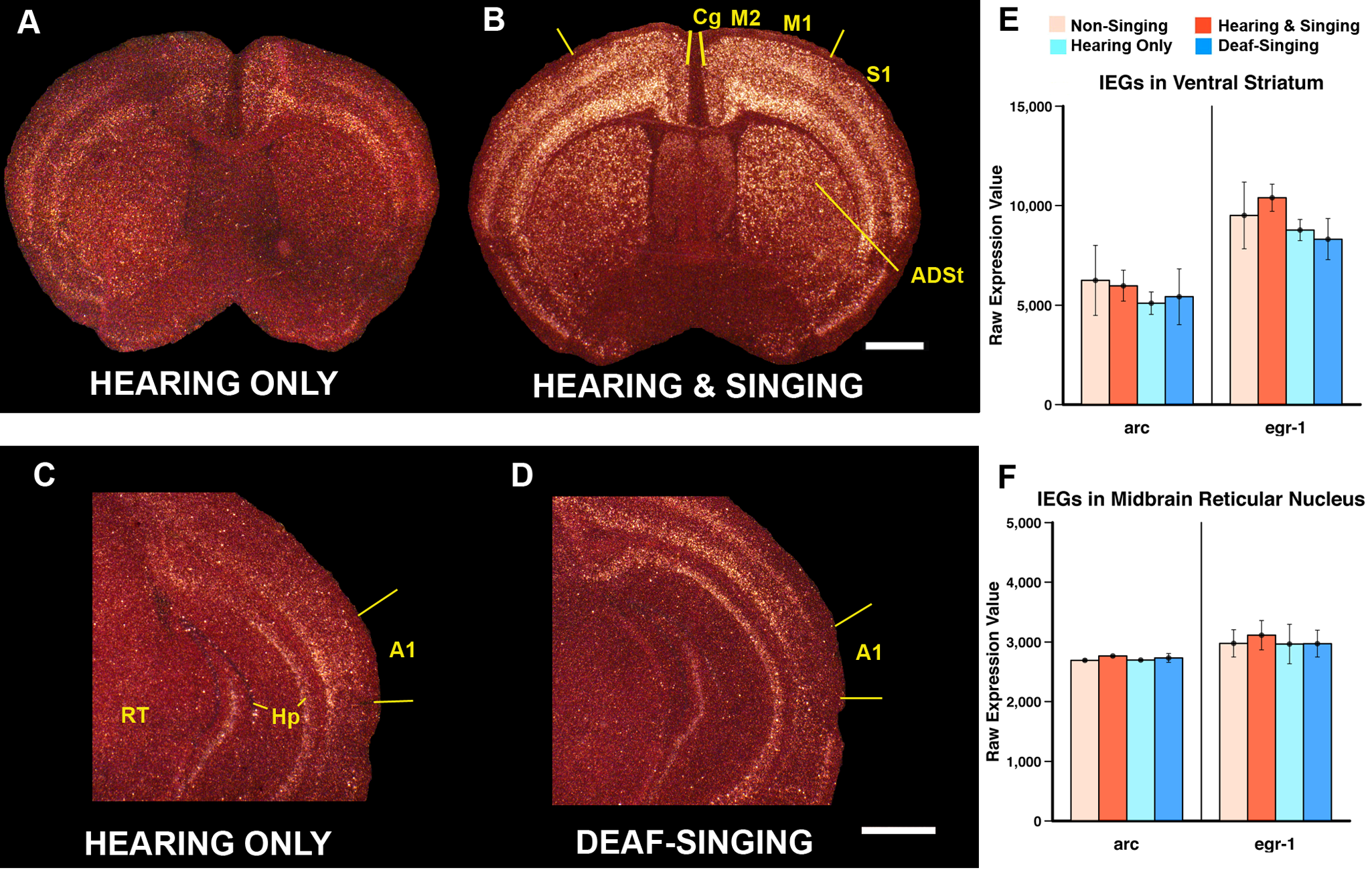

Supplement: Figure S1 — Behavioral-molecular mapping of mouse song system forebrain areas with arc and expression of IEGs in control areas. A–D, Dark-field images of cresyl violet stained (red) coronal brain sections showing singing-induced arc expression (white) in the Hearing & Singing male mice, and reduced expression in the A1 cortex of Deaf-Singing male mice. Sections are adjacent to the same animals shown in Figure 2A–B, D–E. Scale bars, 1 mm. E–F, Raw expression measurements of arc and egr-1 mRNA in the ventral striatum (E) and midbrain reticular (Rt) nucleus (F) showing no difference among the four groups (Kruskal-Wallis H-Test; n = 5 per group; ventral striatum, egr-1: p = 0.3, arc: p>0.5; midbrain reticular nucleus, egr-1: p>0.5, arc: p = 0.070; data are plotted as means ± s.e.m.). These brain areas were used to normalize expression in other brain regions (see methods). Abbreviations are as in Figure 2. (TIF) [file pone.0046610.s015.tif]

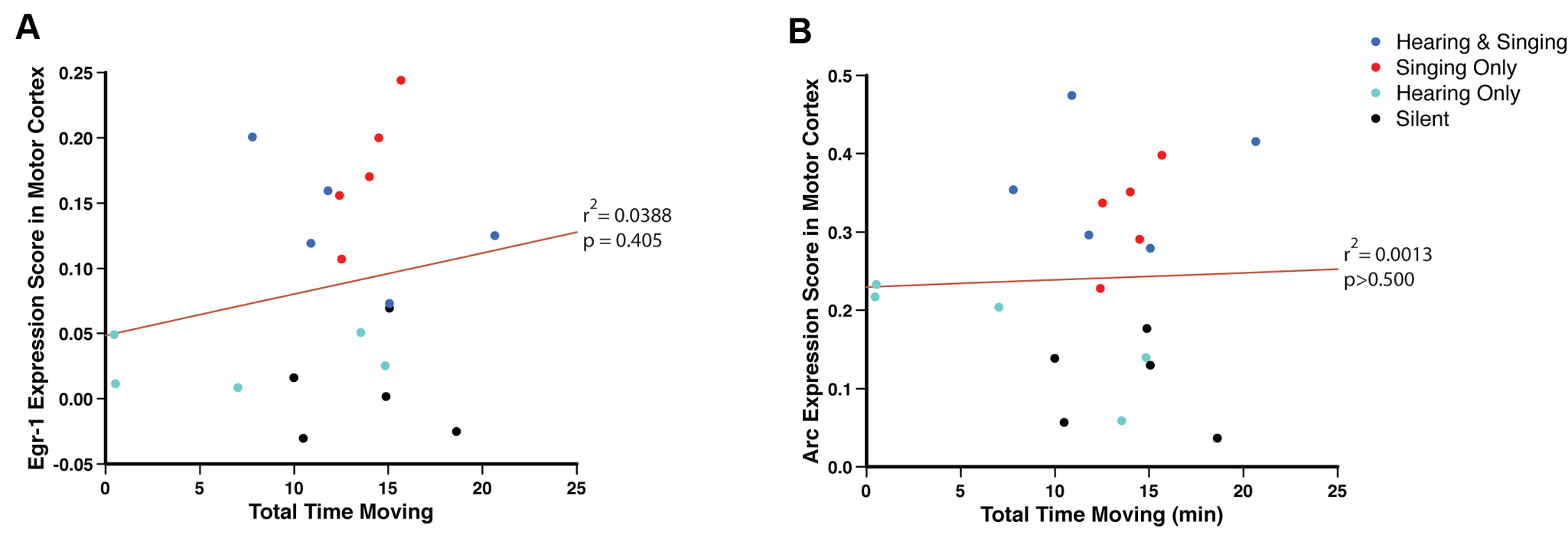

Supplement: Figure S2 — Amount of movement and IEG expression levels in singing active and laryngeal connected M1+M2 region. Shown are linear regressions of arc (A) and egr-1 (B) expression scores (y-axis) relative to the total time spent moving in the cage (x-axis) during the recording session. Movement was scored with the program Annotation by SaySoSoft, and the total time spent making ambulatory back and forth and rotational movement calculated (see methods). Even though there were large differences among some animals, such as two mice in the Hearing Only group that remained relatively still, there was no correlation between the amount of movement and the amount of IEG expression. (TIF) [file pone.0046610.s016.tif]

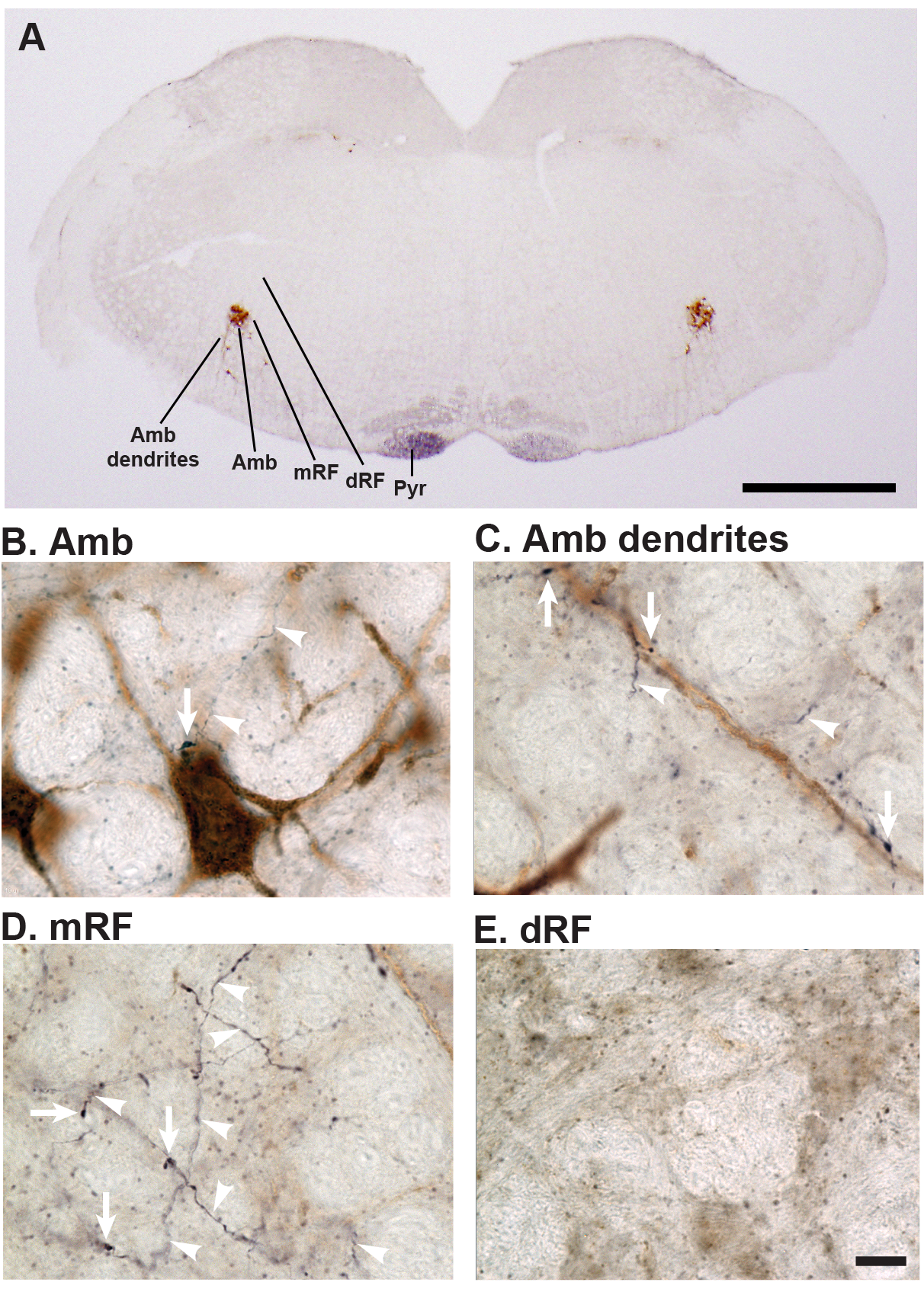

Supplement: Figure S3 — M1 axons in the brainstem. A, Low power view of a coronal brainstem section containing CTb-labeled motor neurons in Amb (brown) from an injection in laryngeal muscles and M1 axons (black) from an injection of BDA into M1 (similar plane of section as in Figure 3A). Only BDA label axons can be seen in the cortico-pyramidal (Pyr) track at this low magnification. Abbreviations: Amb, nucleus ambiguus; Pyr, pyramids; mRF, reticular formation directly medial to Amb; dRF, reticular formation dorsal to Amb. B, High magnification of BDA labeled axon (black) from M1 in Amb that splits near a CTb labeled motor neuron cell body (brown), with one axon branch making a large bouton-like contact (arrow) and the other branch wrapping around the cell body (arrow heads). C, M1 axons (black) running along and near a large Amb motor neuron dendrite that radiates out from Amb. D, Axons (black) in a localized region of the reticular formation directly medial to Amb, where ambiguus motor neuron dendrites pass nearby (brown). E, No axons were seen in the reticular formation further medial and dorsal to Amb. Greyish dots without labeled axons are artifacts of the double labeling protocol. Scale bars: 1 mm for a; 10 µm for B–E. (TIF) [file pone.0046610.s017.tif]

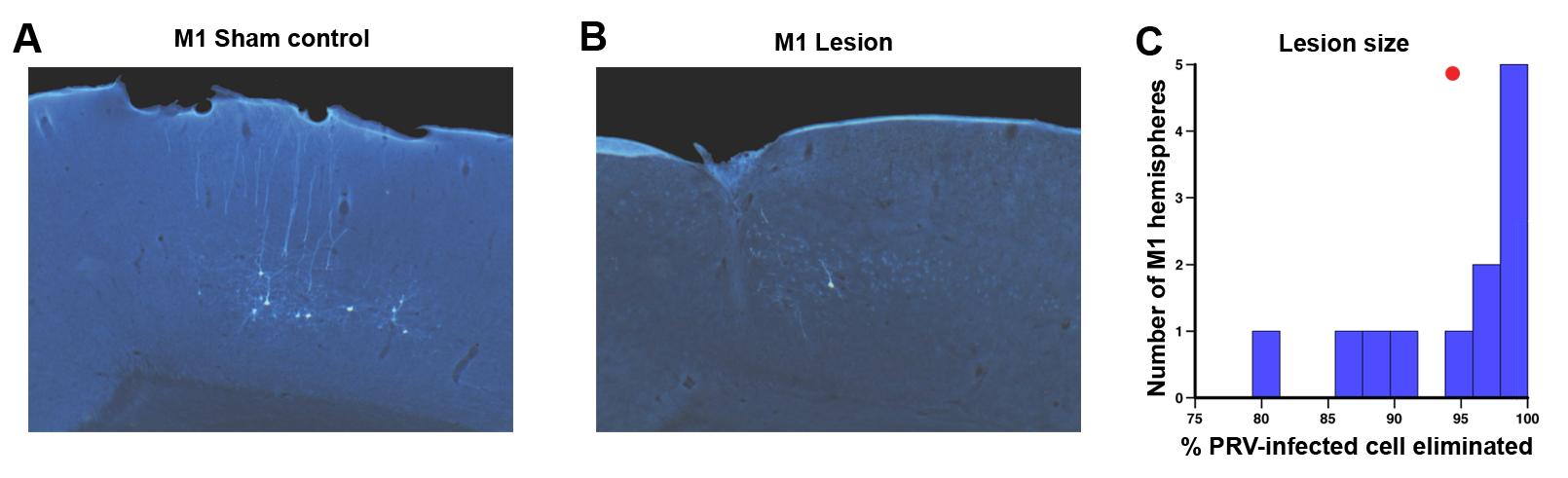

Supplement: Figure S4 — Verification and quantification of M1 lesions. A, eGFP labeled Layer 5 M1 neurons from a PRV-Bartha-eGFP tracer injected in laryngeal muscles of a sham control animal; this result replicates the findings shown in Figure 3C–D, bringing the total number of animals with such backfilled cells to 19. B, Elimination of PRV-Bartha back-traced premotor neurons in M1 following chemical lesions. Scale bars, 1 mm. C, Distribution of lesion sizes based on elimination of PRV-Bartha-eGFP labeled layer 5 pyramidal cells in M1 lesioned animals (12 cerebral hemispheres in 6 mice) relative to an average of sham controls (n = 5 mice). Most lesions eliminated more than 85% of traceable neurons, with a mean lesion size of 94% (red dot). (TIF) [file pone.0046610.s018.tif]

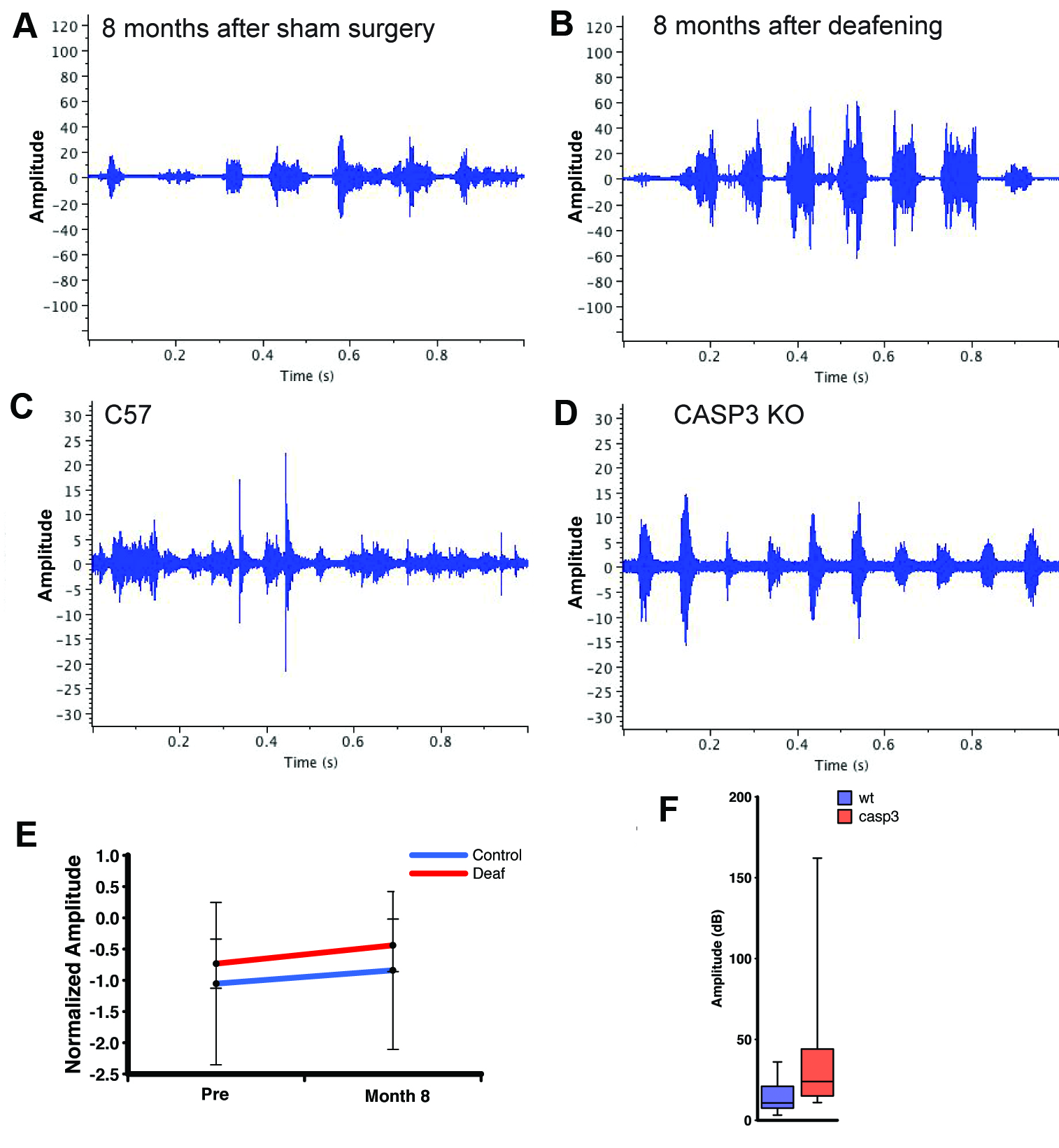

Supplement: Figure S5 — Amplitude of songs from deafened male mice. A–B, Waveforms of song excerpts used to generate the sonograms in Figure 5B and 5F of a hearing-intact sham control and a deafened adult male, respectively. C–D, Waveforms of song excerpts used to generate the sonograms in Figure 5H and 5I , of a wild type C57 and a congentially deaf CASP3 KO male, respectively. The microphones were not saturated during these recordings; saturation causes clipping at the upper and lower ends of the waveforms. E, Normalized amplitudes (SFS) show no differences in sham-operated and deaf adult male mice before and 8 months after surgery (Two-way repeated-measures ANOVA; Treatment: F = 0.203, p>0.5; Recording Session: F = 2.698, p = 0.139; Treatment×Recording Session: F = 0.038; p>0.5; n = 5 per group). F, Normalized amplitude (SFS) show a trend of increased amplitude but the difference is not significant in adult CASP3 KO versus C57 male mice (Student's t-test; p = 0.147; n = 8 C57 and n = 6 CASP3 KO). (TIF) [file pone.0046610.s019.tif]

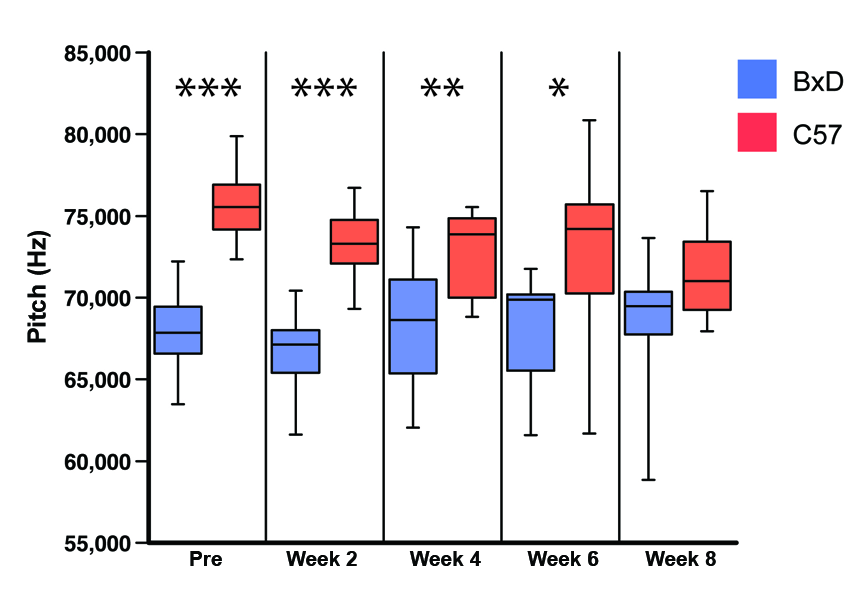

Supplement: Figure S6 — Pooled data for pitch convergence in C57/BxD male pairs housed with either a C57 or BxD female. Group mean pitch of Type A syllables from the songs of C57 and BxD males before and over 8 weeks of cross-strain paired housing, pooled across female strain (BxD female or C57 female). Pitch convergence was also found in the pooled data (* = p<0.05; ** = p<0.01; *** = p<0.001; Student's t-test; Pre: n = 12 C57, n = 12 BxD; Week 2: n = 8 C57, n = 12 BxD; Week 4: n = 6 C57, n = 11 BxD; Week 6: n = 8 C57, n = 11 BxD; Week 8: n = 9 C57, n = 12 BxD). Box plots show the median, 1st and 3rd quartile, and full range. (TIF) [file pone.0046610.s020.tif]

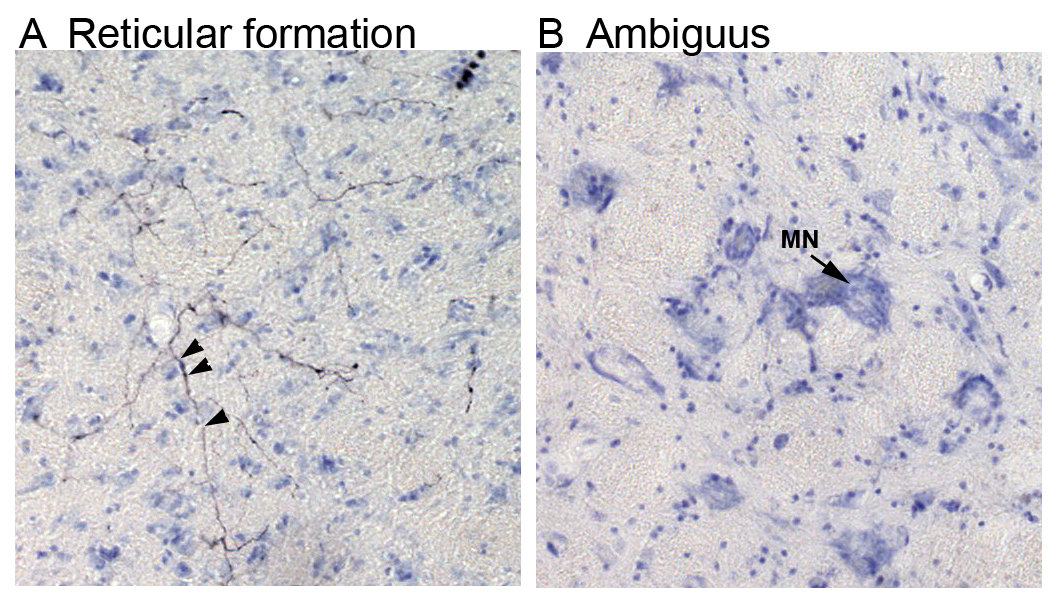

Supplement: Figure S7 — Anterograde tracing from Area 6V in rhesus monkeys. A, BDA labeled axons from Area 6V present in the reticular formation dorsal to nucleus ambiguus. B, Lack of axons in nucleus ambiguus where the motor neurons (MN) are located. Sections are from Kristina Simonyan, and were used for the drawings in a previous study [12]. (TIF) [file pone.0046610.s021.tif]
