# Supplementary material for: Of Mice, Birds, and Men: The Mouse Ultrasonic Song System Has Some Features Similar to Humans and Song-Learning Birds
Source: PLoS One. 2012 Oct 10;7(10):e46610. doi: 10.1371/journal.pone.0046610 (PMC3468587; doi:10.1371/journal.pone.0046610)
Supplement: Text S1 — (DOCX) [file pone.0046610.s022.docx]

SUPPLEMENTAL FILES

### Of mice, birds, and men: the mouse ultrasonic song system has some features similar to humans and song-learning birds

Gustavo Arriaga, Eric P. Zhou & Erich D. Jarvis

Supplementary Text S1:

Beginning with initial discoveries in cats, humans and non-human primates by Kuypers [1,2], multiple investigators have proposed or supported the hypothesis that a direct projection from the laryngeal motor cortex in humans or arcopallium in birds to brainstem vocal motor neurons is associated with the evolution of vocal learning and spoken language. Below is a brief sampling of quotes in reverse chronological order:

(Simonyan & Horwitz, 2011) [3] *It is hypothesized that the location of the laryngeal motor cortex in the primary motor cortex and its direct connections with the brain stem laryngeal motoneurons in humans, as opposed to its location in the premotor cortex with only indirect connections to the laryngeal motoneurons in nonhuman primates, may represent one of the major evolutionary developments in humans toward the ability to speak and vocalize voluntarily*.

(Fitch, Huber & Bugnyar, 2010) [4] *These converging data provide strong support for the Kuypers/Jurgens hypothesis: that direct connections from motor cortex onto the primary motor neurons controlling the vocal apparatus, especially the larynx, are necessary to support complex learned vocalizations. Given these neuroanatomical differences between humans and nonhuman primates, which correlate perfectly with the behavioral lack of vocal learning in other primates, this hypothesis is quite plausible. Songbird data are consistent with these predictions. … Tract tracing studies show that cells in the final songbird motor region in the pallium (homologous to mammalian cortex) indeed send direct monosynaptic projections to the motor neurons controlling the syrinx whose cell bodies lie within the lower brainstem (Wild, 1993, 1997). Such direct connections are not present in suboscine birds, the closest songbird relatives that do not learn their songs.*

(Fischer & Hammerschmidt, 2010) [5] *The most important derived feature in the human lineage regarding the ontogeny of speech appears to be the evolution of the direct pathway from the motor cortex to the motoneurons, enabling volitional control over the oscillations of the vocal folds. Together with the intricate coordination of breathing and articulation, this feature allows for the precise control over speech production.*

(Deacon, 2007) [6] *One of the most likely correlates of allometrically related connection change related to language evolution involves the existence of direct cortical projections to the nucleus ambiguous (the laryngeal control nucleus of the brainstem), which are likely absent in other mammals. … In songbirds … in parrots and possibly hummingbirds there is also a direct projection from the forebrain motor nuclei to brainstem motor systems. This later pattern is perhaps analogous to the direct forebrain (cortical) projections to the vocal motor centers that distinguishes humans.*

(Jarvis, 2004) [7] *I argue that a mutational event that caused descending projections of avian arcopallium neurons to synapse onto nXIIts or mammalian layer 5 neurons of the face motor cortex to synapse onto nucleus ambiguous may be the only major change that is needed to initiate a vocal learning pathway.*

(Okanoya, 2004) [8] *One of the candidates for this question is the direct cortical-medullar pathway for articulation and breathing. In humans, a part of motor cortex directly projects to the medullary nuclei, the nucleus ambiguus, and the nucleus retro-ambiguus. This projection was absent in the squirrel monkey and Jürgens assumes that this projection exists only in humans. Similarly, there is a direct cortical-medullar pathway for articulation and breathing in the zebra finch, a species of songbirds, but a similar projection in pigeons that do not learn to sing [sic]. Thus, this projection exists in the species that show vocal learning while it is absent in the species without vocal learning.*

(Wild, 1997) [9] *Thus, birds and humans have solved the problem of the control of learned vocalizations (speech in humans and song in songbirds) in ways that are in some ways remarkably similar, but which differ with respect to the extent to which the somatic motor system is superimposed on the emotional motor system, this apparently being greater in songbirds than in mammals. Thus, one and the same telencephalic nucleus (RA) can control the output of both the vocal and the respiratory systems…..This may represent a unique evolutionary solution to the problem of coordinative motor control of vocal production, a problem that is also relevant to the control of human speech and song (Gracco 1990).*

(Wild, 1994) [10] *In summary, the brainstem components of the respiratory-vocal system in songbirds and non-songbirds are remarkably similar – except in one the most significant respect, and that is the presence in songbirds of a major projection from the telencephalic nucleus robustus [arcopallium] (RA) to many brainstem nuclear components that together may be regarded as a ‘communication system’ (cf Bass, 1989). At the time of its discovery, nucleus robustus was rightly regarded as a remarkable nucleus by virtue of its direct projections upon a cranial nerve motor nucleus (XIIts; Nottebohm et al 1976), the only other examples of telencephalic projections upon cranial nerve motor nuclei being those in humans and some other primates (Kuypers, 1958; Kuypers and Lawrence, 1967).*

(Kurzinger & Jürgens, 1982) [11] *This discrepancy between man and monkey finds its anatomical correlate in the fact that there is a direct projection from the cortical larynx area to the laryngeal motoneurones in man but not in monkey. The human cortex thus has a more direct control on phonation than that of the monkey. This more direct control is paralleled by an advanced capability of voluntary control of fine vocal fold movements which the monkey lacks almost completely.*

(Kuypers, 1958) [2] *Considered together with the finding in the cat (Kuypers,’58a) these observations indicate that during phylogenetic development the almost exclusively indirect cortico-nuclear connections of lower animals were augmented in primates by direct cortico-nuclear projections, which apparently began to arise especially in the caudal part of the field of origin of the indirect connections. These direct connections subsequently increased in number at the expense of the indirect projections and began to concentrate heavily in the more caudal parts of the pre-central cortex ….. In view of this, the observed quantitative differences consisting of an increase of the direct projection in the higher forms, accompanied by a decrease of the indirect connections, may well constitute the anatomical substratum for what Sherrington called “a greater integration of localized representation of movements in the anthropoids as compared with the monkey” (’23).*

Supplementary Figure Legends

**Figure S1. Behavioral-molecular mapping of mouse song system forebrain areas with arc and expression of IEGs in control areas.**  **A-D**, Dark-field images of cresyl violet stained (red) coronal brain sections showing singing-induced arc expression (white) in the Hearing & Singing male mice, and reduced expression in the A1 cortex of Deaf-Singing male mice. Sections are adjacent to the same animals shown in Figure 2A-B, D-E. Scale bars, 1 mm.  **E-F,** Raw expression measurements of arc and egr-1 mRNA in the ventral striatum (**E**) and midbrain reticular (Rt) nucleus (**F**) showing no difference among the four groups (Kruskal-Wallis H-Test; n=5 per group; ventral striatum, egr-1: p=0.3, arc: p>0.5; midbrain reticular nucleus, egr-1: p>0.5, arc: p=0.070; data are plotted as means ± s.e.m.). These brain areas were used to normalize expression in other brain regions (see methods). Abbreviations are as in Figure 2.

**Figure S2. Amount of movement and IEG expression levels in singing active and laryngeal connected M1+M2 region.**  Shown are linear regressions of arc (**A**) and egr-1 (**B**) expression scores (y-axis) relative to the total time spent moving in the cage (x-axis) during the recording session. Movement was scored with the program Annotation by SaySoSoft, and the total time spent making ambulatory back and forth and rotational movement calculated (see methods). Even though there were large differences among some animals, such as two mice in the Hearing Only group that remained relatively still, there was no correlation between the amount of movement and the amount of IEG expression.

**Figure S3. M1 axons in the brainstem.** **A**, Low power view of a coronal brainstem section containing CTb-labeled motor neurons in Amb (brown) from an injection in laryngeal muscles and M1 axons (black) from an injection of BDA into M1 (similar plane of section as in Figure 3A). Only BDA label axons can be seen in the cortico-pyramidal (Pyr) track at this low magnification. Abbreviations: Amb, nucleus ambiguus; Pyr, pyramids; mRF, reticular formation directly medial to Amb; dRF, reticular formation dorsal to Amb. **B**, High magnification of BDA labeled axon (black) from M1 in Amb that splits near a CTb labeled motor neuron cell body (brown), with one axon branch making a large bouton-like contact (arrow) and the other branch wrapping around the cell body (arrow heads). **C**, M1 axons (black) running along and near a large Amb motor neuron dendrite that radiates out from Amb.  **D,** Axons (black) in a localized region of the reticular formation directly medial to Amb, where ambiguus motor neuron dendrites pass nearby (brown). **E,** No axons were seen in the reticular formation further medial and dorsal to Amb. Greyish dots without labeled axons are artifacts of the double labeling protocol. Scale bars: 1 mm for a; 10 µm for B-E.

**Figure S4. Verification and quantification of M1 lesions. A**, eGFP labeled Layer 5 M1 neurons from a PRV-Bartha-eGFP tracer injected in laryngeal muscles of a sham control animal; this result replicates the findings shown in Figure 3c-d, bringing the total number of animals with such backfilled cells to 19. **B**, Elimination of PRV-Bartha back-traced premotor neurons in M1 following chemical lesions. Scale bars, 1 mm. **C,** Distribution of lesion sizes based on elimination of PRV-Bartha-eGFP labeled layer 5 pyramidal cells in M1 lesioned animals (12 cerebral hemispheres in 6 mice) relative to an average of sham controls (n = 5 mice). Most lesions eliminated more than 85% of traceable neurons, with a mean lesion size of 94% (red dot).

**Figure S5. Amplitude of songs from deafened male mice. A-B,** Waveforms of song excerpts used to generate the sonograms in **Figure 5B and F** of a hearing-intact sham control and a deafened adult male, respectively. **C-D,** Waveforms of song excerpts used to generate the sonograms in **Figure 5H and I,** of a wild type C57 and a congentially deaf CASP3 KO male, respectively. The microphones were not saturated during these recordings; saturation causes clipping at the upper and lower ends of the waveforms. **E**, Normalized amplitudes (SFS) show no differences in sham-operated and deaf adult male mice before and 8 months after surgery (Two-way repeated-measures ANOVA; Treatment: F=0.203, p>0.5; Recording Session: F=2.698, p=0.139; Treatment x Recording Session: F=0.038; p>0.5; n= 5 per group). **F**, Normalized amplitude (SFS) show a trend of increased amplitude but the difference is not significant in adult CASP3 KO versus C57 male mice (Student’s t-test; p=0.147; n=8 C57 and n=6 CASP3 KO ).

**Figure S6. Pooled data for pitch convergence in C57/BxD male pairs housed with either a C57 or BxD female.** Group mean pitch of Type A syllables from the songs of C57 and BxD males before and over 8 weeks of cross-strain paired housing, pooled across female strain (BxD female or C57 female). Pitch convergence was also found in the pooled data (* = p<0.05; ** = p<0.01; *** = p<0.001; Student’s t-test; Pre: n=12 C57, n=12 BxD; Week 2: n=8 C57, n=12 BxD; Week 4: n=6 C57, n=11 BxD; Week 6: n=8 C57, n=11 BxD; Week 8: n=9 C57, n=12 BxD). Box plots show the median, 1^st^ and 3^rd^ quartile, and full range.

**Figure S7. Anterograde tracing from Area 6V in rhesus monkeys.** **A**, BDA labeled axons from Area 6V present in the reticular formation dorsal to nucleus ambiguus. **B**, Lack of axons in nucleus ambiguus where the motor neurons (MN) are located. Sections are from Kristina Simonyan, and were used for the drawings in a previous study [12].

**Supplementary Audio Legends**

All supplementary audios are pitched shifted into the human hearing range, which causes the song production rate to also be slower than normal.

**Audio S1.** Example of a normal adult BxD mouse song (audio corresponds to sonogram of USVs in **Figure 1A**).

**Audio S2.** Example of adult BxD mouse song 1 week before sham brain lesion surgery (audio corresponds to sonogram of USVs in **Figure 4C**).

**Audio S3.** Example of adult BxD mouse song 3 weeks after sham brain lesion surgery (same mouse as **Audio S2;** audio corresponds to sonogram of USVs in **Figure 4D**).

**Audio S4.** Example of adult BxD mouse song 1 week before lesions in laryngeally connected M1 (audio corresponds to sonogram of USVs in **Figure 4E**).

**Audio S5.** Example of adult BxD mouse song 3 weeks after lesions in laryngeally connected M1 (same mouse as **Audio S4;** audio corresponds to sonogram of USVs in **Figure 4F**).

**Audio S6.** Example of adult BxD mouse song 1 month before sham deafening surgery (audio corresponds to sonogram of USVs in **Figure 5A**).

**Audio S7**. Example of adult BxD mouse song 8 months after sham deafening surgery (same mouse as **Audio S6**; audio corresponds to sonograms of USVs in **Figure 5B**)

**Audio S8**. Example of adult BxD mouse song 8 months after sham deafening surgery (same mouse as **Audio S6**; audio corresponds to sonograms of USVs in **Figure 5C**).

**Audio S9.** Example of adult BxD mouse song 1 month before deafening by cochlear removal (audio corresponds to sonogram of USVs in **Figure 5D**).

**Audio S10.** Example of adult BxD mouse song 8 months after deafening by cochlear removal (same mouse as **Audio S8**; audio corresponds to sonograms of USVs in **Figure 5E**).

**Audio S11.** Example of adult BxD mouse song 8 months after deafening by cochlear removal (same mouse as **Audio S8**; audio corresponds to sonograms of USVs in **Figure 5F**).

**Audio S12.** Example of normal adult C57 mouse song (audio corresponds to sonograms of USVs in **Figure 5J**).

**Audio S13.** Example of congenitally deaf CASP3 KO mouse song (audio corresponds to sonogram of USVs in **Figure 5K**).

**Audio S14.** Example of congenitally deaf CASP3 KO mouse song (audio corresponds to sonogram of USVs in **Figure 5L**).

**Supplementary References**

1. Kuypers H (1958) An anatomical analysis of cortico-bulbar connexions to the pons and lower brain stem in the cat. J Anat 92: 198–218.

2. Kuypers H (1958) Some projections from the peri-central cortex to the pons and lower brain stem in monkey and chimpanzee. J Comp Neurol 110: 221–255.

3. Simonyan K, Horwitz B (2011) Laryngeal motor cortex and control of speech in humans. Neuroscientist 17: 197–208. doi:10.1177/1073858410386727.

4. Fitch WT, Huber L, Bugnyar T (2010) Social cognition and the evolution of language: constructing cognitive phylogenies. Neuron 65: 795–814. doi:10.1016/j.neuron.2010.03.011.

5. Fischer J, Hammerschmidt K (2010) Ultrasonic vocalizations in mouse models for speech and socio-cognitive disorders: insights into the evolution of vocal communication. Genes Brain Behav 10: 17–27. doi:10.1111/j.1601-183X.2010.00610.x.

6. Deacon TW (2007) The Evolution of Language Systems in the Human Brain. In: Kaas J, editor. Evolution of Nervous Systems. Amsterdam: Elsevier, Vol. 4. pp. 529–547. Available:http://www.teleodynamics.com/wp-content/PDF/Evolutionlanguagesystems.pdf.

7. Jarvis ED (2004) Learned birdsong and the neurobiology of human language. Ann NY Acad Sci 1016: 749–777.

8. Okanoya K (2004) Functional and structural pre-adaptations to language: insight from comparative cognitive science into the study of language origin. JPN Psychol Res 46: 207–215.

9. Wild JM (1997) Neural pathways for the control of birdsong production. J Neurobiol 33: 653–670.

10. Wild JM (1994) The auditory-vocal-respiratory axis in birds. Brain Behav Evolut 44: 192–209.

11. Kirzinger A, Jürgens U (1982) Cortical lesion effects and vocalization in the squirrel monkey. Brain Res 233: 299–315.

12. Simonyan K, Jürgens U (2003) Efferent subcortical projections of the laryngeal motorcortex in the rhesus monkey. Brain Res 974: 43–59.
